# Effect of an Extract from *Aronia melanocarpa* L. Berries on the Body Status of Zinc and Copper under Chronic Exposure to Cadmium: An In Vivo Experimental Study

**DOI:** 10.3390/nu9121374

**Published:** 2017-12-19

**Authors:** Sylwia Borowska, Małgorzata M. Brzóska, Małgorzata Gałażyn-Sidorczuk, Joanna Rogalska

**Affiliations:** Department of Toxicology, Medical University of Bialystok, Adama Mickiewicza 2C Street, 15-222 Bialystok, Poland; malgorzata.galazyn-sidorczuk@umb.edu.pl (M.G.-S.); joanna.rogalska@umb.edu.pl (J.R.)

**Keywords:** apparent absorption, *Aronia melanocarpa* berries, body status, cadmium, copper, excretion, metabolism, metallothionein, polyphenols, tissue concentrations, zinc

## Abstract

In an experimental model of low-level and moderate environmental human exposure to cadmium (Cd), it was investigated whether the consumption of a polyphenol-rich *Aronia melanocarpa* L. berries (chokeberries) extract (AE) may influence the body status of zinc (Zn) and copper (Cu). The bioelements’ apparent absorption, body retention, serum and tissue concentrations, total pool in internal organs, excretion, and the degree of binding to metallothionein were evaluated in female rats administered 0.1% aqueous AE or/and Cd in their diet (1 and 5 mg/kg) for 3–24 months. The consumption of AE alone had no influence on the body status of Zn and Cu. The extract administration at both levels of Cd treatment significantly (completely or partially) protected against most of the changes in the metabolism of Zn and Cu caused by this xenobiotic; however, it increased or decreased some of the Cd-unchanged indices of their body status. Based on the findings, it seems that rational amounts of chokeberry products may be included in the daily diet without the risk of destroying Zn and Cu metabolisms; however, their potential prophylactic use under exposure to Cd needs further study to exclude any unfavourable impact of these essential elements on the metabolism.

## 1. Introduction

In recent years, there has been growing interest in the beneficial impact on human health of substances naturally occurring in plants which could be included in the diet as functional food, such as polyphenolic compounds [1,2,3,4]. One of the richest sources of polyphenols is *Aronia melanocarpa* L. (*A. melanocarpa*, (*Michx.*) Elliott, *Rosaceae*) berries (chokeberries) [1]. Among the polyphenolic compounds present in these fruits are anthocyanins (cyanidin derivatives), proanthocyanidins, phenolic acids (chlorogenic acid and neochlorogenic acid), and quercetin and its derivatives [1,5]. *Aronia* berries are also rich in other bioactive compounds such as fiber, tannins, pectin, vitamins, minerals, and organic acids [1,6]. Chokeberries are considered to be useful in the protection and treatment of civilisation diseases [1,2,3,4]. Moreover, recent findings from studies in animal models [5,7,8,9,10,11,12] show that polyphenolic compounds, including those present in *A. melanocarpa* berries, may be a promising preventive/therapeutic strategy for xenobiotics, including toxic heavy metals such as cadmium (Cd).

Cd is an environmental pollutant harmful to human health, to which the general population is exposed in everyday life, mainly via food [13,14,15]. Epidemiological studies indicate that chronic, even relatively low-level exposure to this heavy metal, occurring nowadays in industrialised countries, may contribute to the development of numerous unfavourable health outcomes, mainly including renal dysfunction, osteoporosis, cardiovascular system disorders, liver damage, age-related macular degeneration, hearing loss, and cancers [16,17,18,19,20,21]. Moreover, epidemiological evidence shows that there is no safe level of exposure to Cd and it is forecast that the general population’s exposure to this xenobiotic will increase [13].

Taking the above into account, there has been growing interest in the search for effective ways of prevention and treatment of unfavourable health outcomes caused by Cd [8,22,23,24,25,26,27]. In this regard, scientists and nutritionists have been especially focused on dietary products rich in bioelements, vitamins, and polyphenolic compounds [8,22,23,24,25,26,27]. Previously, we revealed that supplementation with zinc (Zn), under moderate and relatively high repeated exposure to Cd, helped decrease the body burden of this toxic metal and protected against some effects of its toxic action [22,23,24,25,26,27]. Nowadays, we are particularly interested in the possibility of using chokeberries in the protection against negative health effects of low-level and moderate chronic exposure to this heavy metal [1,5,7,8,9,10,11]. Using a female rat model of low-level and moderate environmental lifetime human exposure to Cd (1 and 5 mg Cd/kg diets, respectively), we revealed that the consumption of 0.1% aqueous extract from *Aronia* berries (AE) decreased the gastrointestinal absorption and body burden of this toxic metal and increased its urinary excretion [7]. Moreover, the extract also protected against oxidative stress and oxidative changes in the serum and bone tissue [9], bone metabolism disorders and worsening of bone biomechanical properties [5,10], as well as damage to the liver caused by Cd [11] (data in preparation for publication). The beneficial impact of AE may be explained by its antioxidative action and the ability of polyphenols rich in hydroxyl (–OH) groups to complex Cd ions (Cd^2+^) [7,8,9,28]. It is also possible that fiber present in chokeberries may bind Cd ions in the gastrointestinal tract, thus preventing their absorption [6].

Taking into account the above findings, products containing *Aronia* berries seem to be very promising agents for use in the protection against toxic action of Cd; however, their possible prophylactic use needs further studies that allow the evaluation of a wide spectrum of aspects of influence, including the exclusion of any adverse outcomes of their prolonged consumption. Generally, there is no evidence of serious adverse health outcomes of long-term enhanced consumption of polyphenol-rich products in humans. However, it has been noticed that such products may cause antinutritional effects and disorders in bioelements metabolism, as well as affect drug bioavailability and pharmacokinetics [29,30]. It seems crucial to take into account the risk of disorders in the metabolism of necessary elements in the case of prolonged consumption of polyphenol-rich products because of polyphenolic compounds’ ability to bind divalent metals, including bioelements [28,31,32]. Under the conditions of simultaneous exposure to Cd, this is especially important for bioelements interacting with toxic heavy metals, such as Zn and copper (Cu) [22,24,33,34,35,36,37,38].

It is well known that some effects of Cd toxicity result from destroying the metabolisms and biological functions of Zn and Cu [22,24,33,34,35,36,38,39], and that the intake and body status of these bioelements have an influence on the body burden of Cd and its toxicity [22,23,24,25,26,27]. Owing to the presence in chokeberries of compounds capable of binding Zn and Cu [1,6,31,32], and taking into account that changes in the erythrocyte concentrations of both bioelements were noticed in people consuming *Aronia* anthocyanins [29], we found it necessary to investigate whether and how the prolonged consumption of AE, demonstrated by us to have a protective impact under exposure to Cd [5,7,9,10,11], may modify the body status of these elements. Zn and Cu are among the necessary elements playing a key role in the proper functioning of the organism [40,41], and both their deficiency and excessive concentrations in biological fluids and tissues are dangerous for health [41,42].

Taking into account the lower body burden of Cd due to AE consumption [7] and the available data on Cd interactions with Zn and Cu [22,24,33,34,35,36,37,38], we hypothesised that the administration of the extract could, at least partially, prevent Cd-induced disorders in the body status of Zn and/or Cu. However, because AE ingredients may potentially form complexes with Zn and Cu [31,32] and influence the metabolism of these bioelements [29], it cannot be ruled out that the consumption of the extract may have no impact on the body status of these elements or even lead to their deficiency in the organism. Thus, the aim of the present study was to investigate the influence of AE intake on the body status of Zn and Cu in the experimental model of exposure to Cd that we used previously [5,7,9,10,11]. For this purpose, the apparent absorption, retention in the body, and excretion, as well as the serum and tissue concentrations, and the total pool in internal organs were evaluated for both bioelements. The concentration of metallothionein (MT) in the liver, kidney, and duodenal tissue was also determined. Also, the degree of Zn, Cu, and Cd binding to this protein was evaluated, because polyphenols may induce the biosynthesis of MT [43], which plays a key role in the accumulation and detoxification of Cd, as well as in the regulation of Zn and Cu metabolisms [22,33,43,44,45], and due to the fact that this protein is involved in Cd-induced irregularities of the metabolism of both bioelements [22,33]. To the best of our knowledge, such research has not been carried out until now.

## 2. Materials and Methods

### 2.1. Chemicals

Cadmium chloride (CdCl_2_ × 2½ H_2_O) and sodium chloride (NaCl) were purchased from POCh (Gliwice, Poland), while Morbital and heparin were obtained from Biowet (Pulawy, Poland) and Biochemie GmbH (Kundl, Austria), respectively. Trace-pure 65% nitric acid (HNO_3_; Merck, Darmstadt, Germany) and 30% hydrochloric acid (HCl; Merck), as well as stocks of standard solutions of Zn, Cu, and Cd (Sigma, St. Louis, MO, USA) assigned for atomic absorption spectrometry (AAS method) were used. The mixture of palladium and magnesium (as nitrates; Merck) was used as a matrix modifier in Cd analysis. In order to check the analytical quality of metals measurement, the following certified materials were used: Trace Elements Serum L-1 LOT (No. 0903106; Sero, Billingstad, Norway), Trace Elements Urine L-2 LOT (No. 1011645; Sero, Billingstad, Norway), Standard Reference Material Bovine Liver (No. 1577b; National Institute of Standards and Technology, Gaithersburg, MD, USA), Certified Reference Material BCR Pig Kidney (BCR-186; Institute for Reference Materials and Measurements, Geel, Belgium), and Standard Reference Bone Ash (No. 1400; National Institute of Standards and Technology, Gaithersburg, MD, USA). The diagnostic ELISA kit for MT determination was obtained from MyBioSource (San Diego, CA, USA). Ultra-pure water received from a two-way water purification MAXIMA system (ELGA, Bucks, UK) was used in all of the measurements.

### 2.2. Experimental Animals

A total of 192 young (3 to 4 weeks old) female Wistar rats (Crl: WI (Han)) purchased from the certified Laboratory Animal House (Brwinów, Poland) were used. Throughout the experiment, the animals were housed in controlled conventional conditions (temperature 22 ± 2 °C, relative humidity 50 ± 10%, 12/12 h light-dark cycle). They were maintained in stainless-steel cages, four animals in each, and had free access to drinking water and food during the whole experiment. Throughout the first 3 months of the study, all rats received the Labofeed H diet (breeding diet ensuring the proper growth and development of young animals; Label Food ‘Morawski’, Kcynia, Poland), following which they were fed with the Labofeed B diet (maintenance diet). According to the manufacturer, the Labofeed H diet contained 210 mg Zn/kg and 33 mg Cu/kg, whereas the Labofeed B diet contained 150 mg Zn/kg and 25 mg Cu/kg. The concentrations of Zn and Cu determined (using the AAS method) in our laboratory in the Labofeed H diet reached 202.1 ± 2.44 mg/kg (mean ± standard deviation—SD) and 29.62 ± 0.51 mg/kg, respectively, while in the Labofeed B diet they were 143.3 ± 2.6 mg/kg and 22.91 ± 1.37 mg/kg, respectively.

### 2.3. Experimental Protocol

The research protocol was approved by the Local Ethics Committee for Animal Experiments in Bialystok, Poland (approval No. 60/2009 of 21 September 2009 and approval No. 80/2015 of 9 June 2015) and performed according to the ethical principles and institutional guidelines, as well as the international Guide for the Use of Animals in Biomedical Research.

The experimental model has been described in detail in our previous reports [5,7,9,10]. In brief, the rats were randomly divided into six groups (32 animals each) receiving Cd and/or AE or not, as described in Table 1. One group received 0.1% AE alone, two groups were given Cd alone in the Labofeed diets containing 1 and 5 mg Cd/kg, and the next two groups received AE during the whole course of Cd exposure (for up to 24 months). The control group drank redistilled water without AE addition and was fed with the standard Labofeed diets without Cd (Table 1). The daily intake of Cd at particular levels of treatment and the intake of AE throughout the experiment did not differ regardless of whether they were administered alone or in conjunction (Table 1).

Because the diet, being the main source of the general population’s exposure to Cd [13], is also a source of bioelements and various biologically active compounds, including polyphenols, toxic (Cd) and essential (Zn and Cu) metals and polyphenolic compounds can interact with one another in the gastrointestinal tract, thus affecting their own bioavailability and absorption, and thus also the body status [29,43,46,47]. That is why, in our experimental model, Cd was administered in the diet at concentrations that simulated the conditions reflecting the general population’s exposure [14,16,17,18,19,20,48,49]. The measurements of Cd concentration in the blood and urine of the rats receiving the diets containing 1 and 5 mg Cd/kg (0.113–0.324 μg/L and 0.085–0.354 μg/g creatinine, and 0.584–1.332 μg/L and 0.284–0.820 μg/g creatinine, respectively) [7] confirmed that the used experimental model corresponds well with human environmental exposure to this toxic metal in industrialised countries [20,48,49]. Since women are characterised by higher gastrointestinal absorption of Cd and are more susceptible to its toxicity [50], the study was performed in a female rat model.

During the experiment, there were no statistically significant differences in the consumption of food and drinking water, or body weight gain among the experimental groups [7]. Moreover, no unfavourable health outcomes were observed; however, three animals died between the 18th and 24th month of the experiment (one case of death in each of the AE, Cd_1_, and Cd_5_ groups) [7].

In the last week of the 3rd, 10th, 17th, and 24th month of the experiment, a 5-day balance study was performed. For this purpose, the rats (eight animals of each group at each time point, except for seven animals in some groups after 24 months) were placed individually in metabolic cages and 24-hour urine and faeces were collected on 5 consecutive days. During this time, the animals had free access to food and drinking water (with or without Cd and AE, depending on the experimental group), the consumption of which was monitored. The urine and faeces were removed from the metabolic cages every 24 h and stored for further analysis. The urine was centrifuged (MPW-350R centrifugator, Medical Instruments, Warsaw, Poland) immediately after collection and its volume was recorded. After the balance study, the rats were deprived of food overnight and then they were subjected to anaesthesia (Morbital, 30 mg/kg b.w., intraperitoneally). The whole blood was taken by cardiac puncture with and without anticoagulant (heparin). Stomach, duodenum, liver, kidneys, spleen, heart, brain, femur, and femoral muscle were dissected. The content of the stomach and duodenum was immediately removed by multiple rinsing with ice-cold physiological saline (0.9% NaCl). The dissected soft tissues, after rinsing with ice-cold 0.9% NaCl, were gently dried on the filter paper, whereas the femurs were cleaned of all adherent soft tissues. Next, they were weighed with an analytical balance (OHAUS^®^, Nanikon, Switzerland; accuracy to 0.0001 g). The biological material which was not used immediately was stored frozen at −70 °C until assayed.

### 2.4. Determination of Zn and Cu in Biological Fluids, Tissues, and Faeces

The 5-day faeces, after drying to constant weight, was crumbled, and three representative samples for each rat were collected for analysis. Known-weight slices of the liver, kidney (left), spleen, heart, brain, femoral muscle, stomach, and duodenum, as well as the samples of faeces were wet-digested with a mixture of trace-pure concentrated HNO_3_ and HCl using a microwave system (Multiwave, Anton Paar GmbH, Graz, Austria) as reported [7]. Next, the excess of acids was evaporated by slightly warming up the samples and the preparations were diluted with ultra-pure water. Bone slices (0.1–0.2 g) obtained from the distal epiphysis (trabecular bone region) and diaphysis (compact bone region) of the femur (left), after rinsing with ultra-pure water (to eliminate the removable bone marrow), drying (to receive dry bone weight), and ashing, were wet-digested with trace-pure 65% HNO_3_, and then diluted with ultra-pure water [7]. The samples of the serum and 24-h urine (representative samples from the 5-day balance study) were diluted with 0.5% HNO_3_.

The concentrations of Zn and Cu in such preparations of the serum, urine, faeces, soft tissues, and bone tissue, except for Cu in the bone tissue, were determined (after appropriate dilution if necessary) by the flame AAS method (F AAS; atomisation in an air–acetylene burner). The concentration of Cu in the bone preparations was measured by the flameless AAS method with electrothermal atomisation in a graphite furnace (GF AAS). An atomic absorption spectrophotometer model Z-5000 (Hitachi, Tokyo, Japan) equipped with cathode lamps of Zn and Cu (resonance lines of 213.8 nm and 324.8 nm, respectively; Photron, Narre Warren, Australia) was used. The limit of detection of Zn and Cu for the F AAS method was 8.9 μg/L and 21 μg/L, respectively, while the limit of Cu detection for the GF AAS method was 0.355 μg/L. Zn and Cu concentrations in the soft tissues are expressed as per gram of wet tissue weight, while in the bone tissue they are expressed as per gram of dry bone weight.

In order to check the analytical quality of Zn and Cu measurements, the concentrations of both metals in the certified reference serum, liver, kidney, bone, and urine were measured. The concentrations determined in our laboratory agreed exactly with the certified values, and the recovery ranged from 95% to 103% for Zn and from 96% to 105% for Cu, whereas the precision of measurements, expressed as a coefficient of variation (CV), was <3.3% for Zn and <9.4% (<7.9% for the GF AAS and <9.4% for the F AAS) for Cu (detailed results are presented in Table S1).

### 2.5. Estimation of the Bioavailability and Retention of Zn and Cu in the Body 

The bioavailability of Zn and Cu was evaluated based on their apparent absorption expressed as the absorption index (Abs_Zn_ and Abs_Cu_ (%)) calculated from the following equation: Abs_Me_ = (I_Me_ − FE_Me_)/I_Me_ × 100%, where Me means Zn or Cu, I_Me_ is the mean daily intake of Zn or Cu via food during the 5-day balance study, and FE_Me_ is the mean amount of Zn or Cu_excreted daily with faeces during the 5-day study [51].

The mean daily body retention of Zn or Cu (R_Me_ (%)) was calculated as the difference between the mean daily intake of a particular bioelement (I_Me_) during the 5-day balance study and the mean amount of the element excreted daily with faeces (FE_Me_) and urine (UE_Me_) during this time, according to the following formula: R_Me_ = [I_Me_ − (FE_Me_ + UE_Me_)]/I_Me_ × 100% [51].

### 2.6. Calculation of the Total Pool of Zn and Cu in Internal Organs

The total pool of Zn or Cu in internal organs was calculated as the sum of the content of a particular element in organs such as liver, spleen, heart, brain, and both kidneys. Because the concentrations of Zn and Cu were determined only in the left kidney, to evaluate the total content of these bioelements in both kidneys it was assumed that their concentrations in the right kidney were the same as those in the left one. Zn and Cu concentrations in the right kidney were calculated by the multiplication of these metal concentrations in the left organ and the weight of the right organ.

The total content of a bioelement in particular internal organs, and especially its total pool in these organs, are better parameters to reflect the body status of the bioelement than its content or concentration in particular organs. Differences in the weight of internal organs may mask changes in the element concentration in these organs, especially in the case of a very slight change. The estimation of the total pool of a bioelement in internal organs may allow the detection of a slight change (shortage or growth) of the bioelement status in the body, which does not yet result in a decrease in this element’s content or concentration in particular organs.

### 2.7. Determination of Cd in the Duodenum

Cd concentration in the wet-digested slices of the duodenal tissue (prepared as described in Section 2.4) was determined using the GF AAS method (detection limit—0.018 μg Cd/L; Hitachi Z-5000 spectrophotometer), as previously reported for this metal assay in other tissues and biological fluids of these rats [7]. Cd concentration determined by us in the certified reference soft tissues (liver—0.513 ± 0.024 μg/g and kidney—2.66 ± 0.14 μg/g; mean ± SD) agreed exactly with the certified values (0.50 ± 0.03 μg/g, 2.71 ± 0.15 μg/g, respectively). The recovery of Cd was 103% and 98%, respectively, and the CV was <4.7%.

### 2.8. Determination of MT Concentration in the Liver, Kidney, and Duodenum, as Well as Estimation of the Degree of Zn, Cu, and Cd Binding to This Protein 

The concentration of MT in the aliquots of 10% homogenates of the liver and kidney, and 20% homogenates of the duodenal tissue (prepared in 0.9% NaCl) was measured colourimetrically using a Rat Metallothionein ELISA Kit. The CV for MT measurements in the kidney, liver, and duodenum was <7.6%, 7.4%, and 5.5%, respectively.

In order to be stable, MT needs to have all metal-binding sites saturated (1 mole of MT needs to contain 7 moles of divalent metals Zn and/or Cd, and 10–12 moles of Cu in +1 valence state) [33,37]. Thus, an increase in the concentrations of Cd and MT in the kidney, liver, and duodenum may be accompanied with a simultaneous increase in the concentrations of Zn and Cu, as well as changes of the pools of these bioelements bound and unbound to MT [22,37]. A decrease in the non-MT-bound pool of Zn or Cu in the duodenal tissue or internal organs may result in hampered gastrointestinal absorption of these bioelements or their insufficient availability to ensure the proper course of physiological processes dependent on them. That is why, studying the impact of the administration of AE on the body status of Zn and Cu in the case of exposure to Cd, it was very important to evaluate the pools of MT-bound and MT-unbound elements in the kidney, liver, and duodenum.

Based on the concentrations of Zn, Cu, Cd, and MT in the kidney, liver, and duodenum, the ratio of actual metal concentration (Me) to the theoretical maximum concentration of MT-bound metal (Me-MT) was calculated according to the following formula: Me/Me-MT = Zn/(MT × 7) + Cd/(MT × 7) + Cu/(MT × 12), where Zn, Cu, Cd, and MT are the concentrations (nmol/g wet tissue) of particular metals and MT [22,37]. To estimate the amount of theoretical maximum MT saturation with metals, MT concentration was multiplied by 7 in the case of Zn and Cd (7 moles of divalent metals, including Zn and Cd, can bind to 1 mole of MT) and by 12 in the case of Cu (10–12 moles of Cu(I) can bind to 1 mole of MT) [22,37]. The values of the ratios of Zn/(MT × 7), Cu/(MT × 12), and Cd/(MT × 7) reflect MT saturation with Zn, Cu, and Cd, respectively, in addition to the pool of MT-unbound particular elements, whereas the ratio of Me/Me-MT reflects this protein saturation with all these metals (and the pool of non-MT bound metals). Theoretically, if the value of the Me/Me-MT ratio is lower than 1, all metals can be bound by MT. When the ratio is higher than 1, there exists the pool of non-MT-bound metals because MT cannot bind them further due to the saturation of its all metal-binding sites. The pool of non-MT-bound metals increases together with the growing ratios of Zn/(MT × 7), Cu/(MT × 12), Cd/(MT × 7), and Me/Me-MT. An increase in the Zn/(MT × 7), Cu/(MT × 12), Cd/(MT × 7), or Me/Me-MT ratio compared to the control group or any other experimental group indicates a rise in the pool of MT-unbound metals (Zn, Cu, Cd, or all of the metals, respectively), while a decrease in these ratios reflects a drop in the pool of non-MT-bound metals. For the calculation of MT saturation with Cd, the concentration of this metal in the liver and kidney previously determined in these animals (Table S2) [7] was used. Only non-MT-bound Cd present in tissues may exert toxic action [22,33,37].

### 2.9. Statistical Analysis

The data are expressed as a mean ± standard error (SE) for eight rats after 3, 10, 17, and 24 months, except for seven animals in groups AE, Cd_1_, and Cd_5_ after 24 months and eight to 32 animals in the case of the daily intake of Zn and Cu presented in Table 2.

A one-way analysis of variance (ANOVA) was applied to determine whether there were statistically significant (*p* < 0.05) differences among the six experimental groups, and then Duncan’s multiple range post hoc test was performed for comparison between individual groups and to determine which two means differed (*p* < 0.05). In tables and figures, statistically significant differences in relation to the control group, the respective group receiving Cd alone (Cd_1_ + AE vs. Cd_1_ and Cd_5_ + AE vs. Cd_5_), and the respective group exposed to the 1 mg Cd/kg diet alone or with AE (Cd_5_ vs. Cd_1_ and Cd_5_ + AE vs. Cd_1_ + AE) are marked. In the case when the Duncan’s multiple range test revealed any influence of the co-administration of Cd and AE on the investigated parameter, a two-way analysis of variance (ANOVA/MANOVA, test F) was conducted so as to discern a possible interactive and independent impact of Cd and AE on this parameter. F values with *p* < 0.05 were recognised as statistically significant. All of the calculations were performed using the Statistica package (StatSoft, Tulsa, OK, USA).

## 3. Results

### 3.1. Daily Intake of Zn and Cu

The mean daily intake of Zn via the Labofeed diet in the control group during the 5-day balance study in the last week of the 3rd, 10th, 17th, and 24th month reached 5.182 ± 0.073 mg/24 h, 2.994 ± 0.087 mg/24 h, 3.356 ± 0.061 mg/24 h, and 4.196 ± 0.071 mg/24 h, respectively, whereas the intake of Cu reached 0.814 ± 0.011 mg/24 h, 0.499 ± 0.015 mg/24 h, 0.559 ± 0.010 mg/24 h, and 0.699 ± 0.012 mg/24 h, respectively (Table S3). Because the content of Zn and Cu in the Labofeed H diet administered during the first 3 months was higher than that in the Labofeed B diet (used from the beginning of the 4th month), the mean daily intake of these elements in all groups during this period of the experiment (Table 2), including the 5-day balance study in the last week of the 3rd month (Table S3), was higher than thereafter (2.1–2.7 times and 17–73%, respectively, for Zn, and 2.1–2.7 times and 10–63%, respectively, for Cu). The daily intake of Zn and Cu via the Labofeed diet at particular time points did not differ between the experimental groups (Table 2 and Table S3).

The Labofeed diet was the main source of Zn and Cu for the experimental animals. Because of very low concentrations of Zn (1.39 ± 0.04 μg/L) and Cu (0.803 ± 0.065 μg/L) in the 0.1% AE, the daily intake of these bioelements via the extract consumption was negligible compared to their intake with the diet. The mean intake of Zn via AE consumption throughout the 24-month study ranged from 0.057 to 0.060 μg/24 h (0.19–0.20 μg/kg b.w.), whereas the intake of Cu reached 0.033–0.035 μg/24 h (0.11–0.12 μg/kg b.w.), regardless of whether the extract was administered alone or with Cd.

The above data show that the total daily intake of Zn and Cu from all sources during particular experimental periods did not differ between the experimental groups, confirming the usefulness of the experimental model to estimate the influence of AE and/or Cd consumption on the body status of these bioelements.

### 3.2. Effect of AE on the Body Status of Zn under Exposure to Cd

#### 3.2.1. Zn Apparent Absorption, Retention in the Body, and Its Faecal and Urinary Excretion 

**Figure 1:**
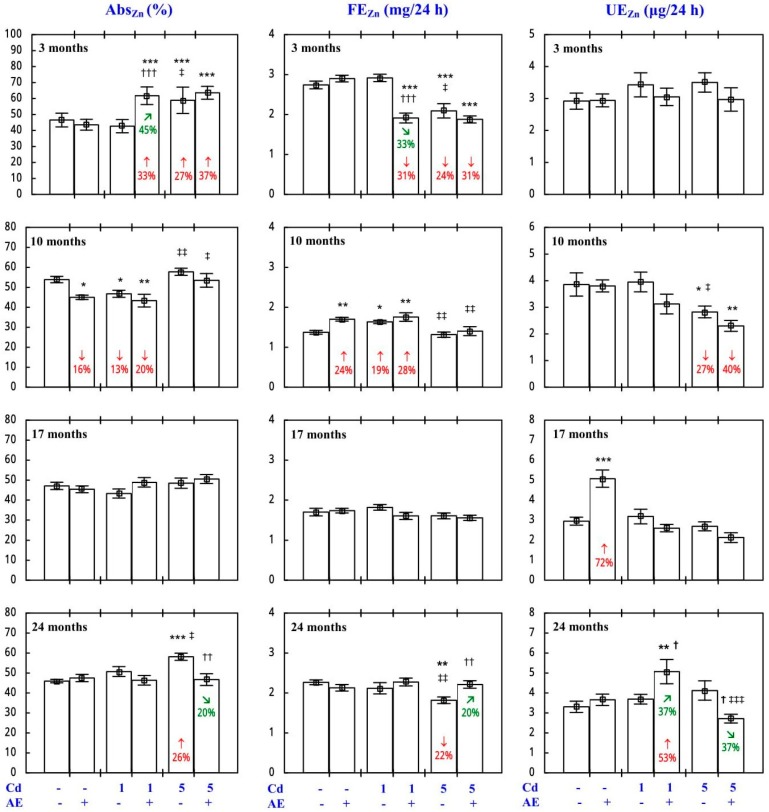
The apparent absorption of zinc (Abs_Zn_) and its daily faecal (FE_Zn_) and urinary excretion (UE_Zn_) in particular experimental groups. The rats received cadmium (Cd) in their diet at the concentration of 0, 1, and 5 mg/kg and/or 0.1% extract from the berries of *Aronia melanocarpa* (AE; “+”, received; “-”, not received). Data represent mean ± SE for eight rats (except for seven animals in the AE, Cd_1_, and Cd_5_ groups after 24 months). Statistically significant differences (ANOVA, Duncan’s multiple range test): * *p* < 0.05, ** *p* < 0.01, *** *p* < 0.001 vs. control group; ^†^ *p* < 0.05, ^††^ *p* < 0.01, ^†††^ *p* < 0.001 vs. respective group receiving Cd alone; ^‡^ *p* < 0.05, ^‡‡^ *p* < 0.01, ^‡‡‡^ *p* < 0.001 vs. respective group receiving the 1 mg Cd/kg diet (alone or with AE) are marked. Numerical values in bars indicate percentage changes compared to the control group (↓, decrease; ↑, increase) or the respective group receiving Cd alone (↘, decrease; ↗, increase).

Because of the low daily urinary excretion of Zn (UE_Zn_; Figure 1), the body retention of this bioelement (Ret_Zn_) in some experimental groups reached almost the same values as the Abs_Zn_ (Figure 1), and thus it is presented in Figure S1 (the same refers to Cu).

The Abs_Zn_ and Ret_Zn_ in the control animals reached 46–54% (Figure 1 and Figure S1). The administration of AE alone had no impact on the Abs_Zn_ and Ret_Zn_, and Zn excretion (FE_Zn_ and UE_Zn_), with only a few exceptions (Figure 1 and Figure S1; Supplementary Material—Zinc 1).

In the rats exposed to the 1 mg Cd/kg diet, the only change in the Abs_Zn_ and Ret_Zn_ was their decrease after 10 months, whereas under exposure to the 5 mg Cd/kg diet these variables increased after 3 and 24 months (Figure 1 and Figure S1). The FE_Zn_ was affected at the same time points as the Abs_Zn_ and Ret_Zn_, but in the opposite direction. The only Cd-induced change in the UE_Zn_ was its decrease after 10 months of the application of the 5 mg Cd/kg diet (Figure 1).

The consumption of AE during the treatment with Cd modified this heavy metal influence on the Abs_Zn_, Ret_Zn_, FE_Zn_, and UE_Zn_ (Figure 1 and Figure S1; Supplementary Material—Zinc 1). The impact depended on the duration of the co-administration of *Aronia* extract and Cd and the level of exposure to this xenobiotic (Figure 1 and Figure S1). The use of the extract for 24 months completely prevented the Cd-induced increase in the Abs_Zn_ and Ret_Zn_, as well as the decrease in the FE_Zn_ (Figure 1). The ANOVA/MANOVA analysis (Table S4) revealed that the modifying effect of AE consumption during the exposure to Cd on the Abs_Zn_, Ret_Zn_, FE_Zn_, and UE_Zn_, including its protective action against Cd impact on the values of these variables, was the result of independent action of the extract ingredients (F = 5.88–27.0, *p* < 0.05–0.001) and/or their interaction with this heavy metal (F = 7.45–48.7, *p* < 0.05–0.001). However, in the case of the Ret_Zn_ in the Cd_5_ + AE group after 24 months, in spite of the total protection offered by AE against the Cd-induced increase in the value of this variable, neither a statistically significant independent impact of AE nor its interaction with Cd were observed (Table S4).

#### 3.2.2. Zn Concentration in the Serum and Tissues 

**Figure 2:**
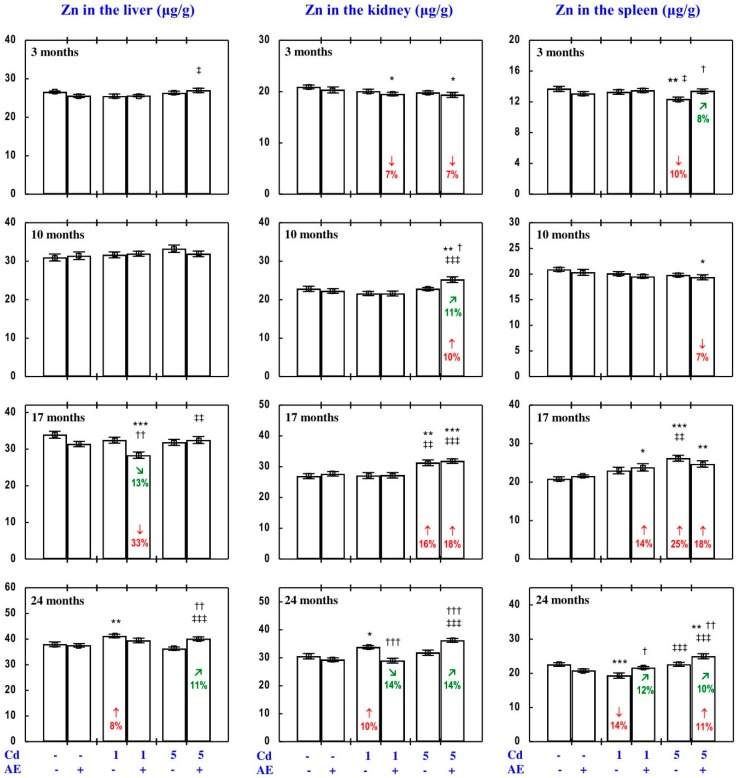
Zinc (Zn) concentration in the liver, kidney, and spleen in particular experimental groups. The rats received cadmium (Cd) in their diet at the concentration of 0, 1, and 5 mg/kg and/or 0.1% extract from the berries of *Aronia melanocarpa* (AE; “+”, received; “-”, not received). Data represent mean ± SE for eight rats (except for seven animals in the AE, Cd_1_, and Cd_5_ groups after 24 months). Statistically significant differences (ANOVA, Duncan’s multiple range test): * *p* < 0.05, ** *p* < 0.01, *** *p* < 0.001 vs. control group; ^†^ *p* < 0.05, ^††^ *p* < 0.01, ^†††^ *p* < 0.001 vs. respective group receiving Cd alone; ^‡^ *p* < 0.05, ^‡‡^ *p* < 0.01, ^‡‡‡^ *p* < 0.001 vs. respective group receiving the 1 mg Cd/kg diet (alone or with AE) are marked. Numerical values in bars indicate percentage changes compared to the control group (↓, decrease; ↑, increase) or the respective group receiving Cd alone (↘, decrease; ↗, increase).

**Figure 3:**
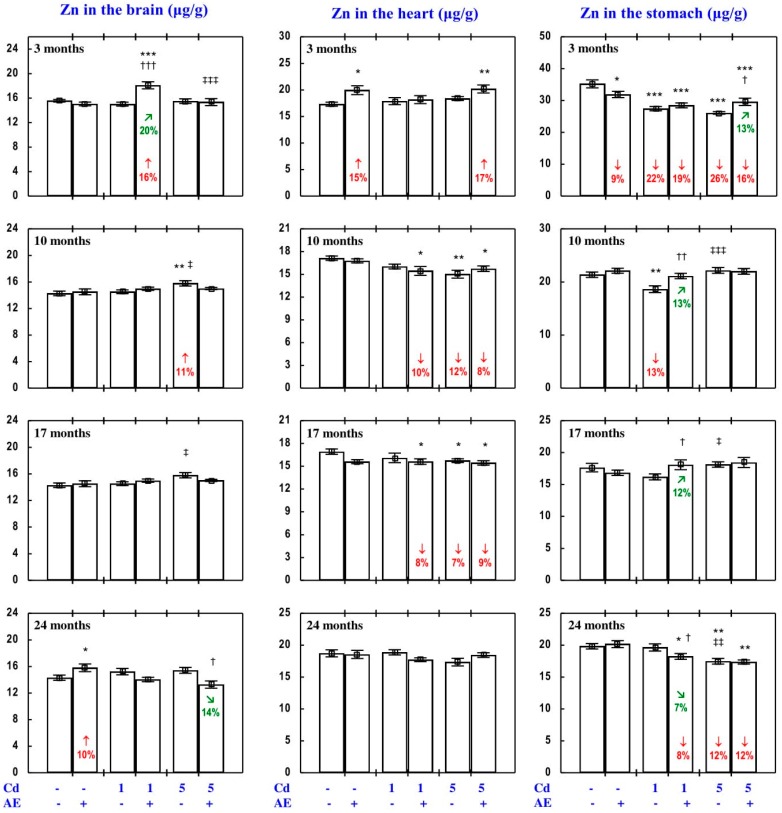
Zinc (Zn) concentration in the brain, heart, and stomach in particular experimental groups. The rats received cadmium (Cd) in their diet at the concentration of 0, 1, and 5 mg/kg and/or 0.1% extract from the berries of *Aronia melanocarpa* (AE; “+”, received; “-”, not received). Data represent mean ± SE for eight rats (except for seven animals in the AE, Cd_1_, and Cd_5_ groups after 24 months). Statistically significant differences (ANOVA, Duncan’s multiple range test): * *p* < 0.05, ** *p* < 0.01, *** *p* < 0.001 vs. control group; ^†^ *p* < 0.05, ^††^ *p* < 0.01, ^†††^ *p* < 0.001 vs. respective group receiving Cd alone; ^‡^ *p* < 0.05, ^‡‡^ *p* < 0.01, ^‡‡‡^ *p* < 0.001 vs. respective group receiving the 1 mg Cd/kg diet (alone or with AE) are marked. Numerical values in bars indicate percentage changes compared to the control group (↓, decrease; ↑, increase) or the respective group receiving Cd alone (↘, decrease; ↗, increase).

**Figure 4:**
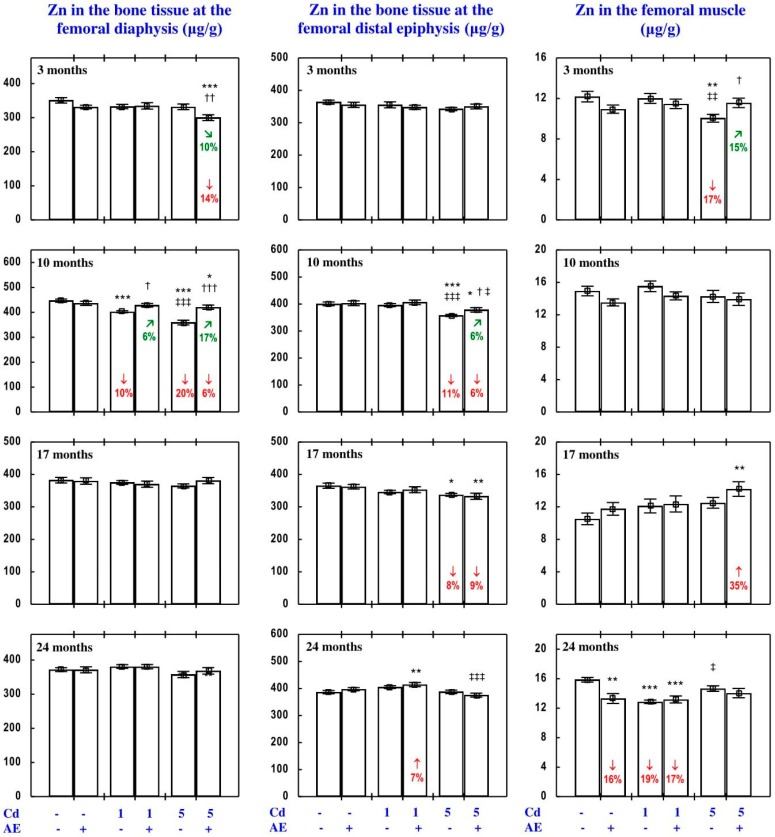
Zinc (Zn) concentration in the bone tissue and femoral muscle in particular experimental groups. The rats received cadmium (Cd) in their diet at the concentration of 0, 1, and 5 mg/kg and/or 0.1% extract from the berries of *Aronia melanocarpa* (AE; “+”, received; “-”, not received). Data represent mean ± SE for eight rats (except for seven animals in the AE, Cd_1_, and Cd_5_ groups after 24 months). Statistically significant differences (ANOVA, Duncan’s multiple range test): * *p* < 0.05, ** *p* < 0.01, *** *p* < 0.001 vs. control group; ^†^ *p* < 0.05, ^††^ *p* < 0.01, ^†††^ *p* < 0.001 vs. respective group receiving Cd alone; ^‡^ *p* < 0.05, ^‡‡^ *p* < 0.01, ^‡‡‡^ *p* < 0.001 vs. respective group receiving the 1 mg Cd/kg diet (alone or with AE) are marked. Numerical values in bars indicate percentage changes compared to the control group (↓, decrease; ↑, increase) or the respective group receiving Cd alone (↘, decrease; ↗, increase).

**Figure 5:**
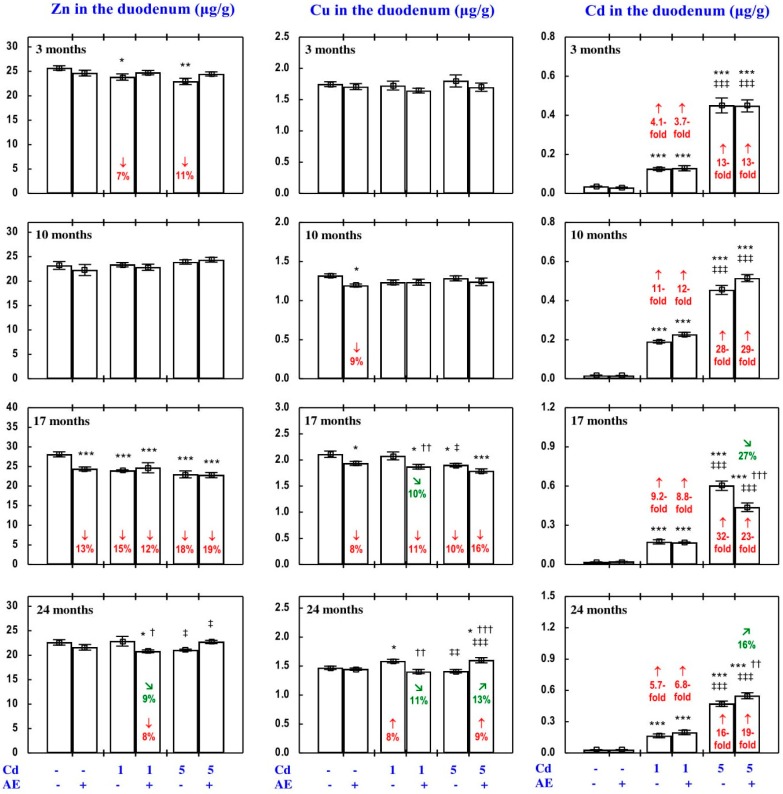
Zinc (Zn), copper (Cu), and cadmium (Cd) concentrations in the duodenum in particular experimental groups. The rats received Cd in their diet at the concentration of 0, 1, and 5 mg/kg and/or 0.1% extract from the berries of *Aronia melanocarpa* (AE; “+”, received; “-”, not received). Data represent mean ± SE for eight rats (except for seven animals in the AE, Cd_1_, and Cd_5_ groups after 24 months). Statistically significant differences (ANOVA, Duncan’s multiple range test): * *p* < 0.05, ** *p* < 0.01, *** *p* < 0.001 vs. control group; ^†^ *p* < 0.05, ^††^ *p* < 0.01, ^†††^ *p* < 0.001 vs. respective group receiving Cd alone; ^‡^ *p* < 0.05, ^‡‡^ *p* < 0.01, ^‡‡‡^ *p* < 0.001 vs. respective group receiving the 1 mg Cd/kg diet (alone or with AE) are marked. Numerical values in bars (or above the bars) indicate percentage changes or factors of changes compared to the control group (↓, decrease; ↑, increase) or the respective group receiving Cd alone (↘, decrease; ↗, increase).

**Figure 6:**
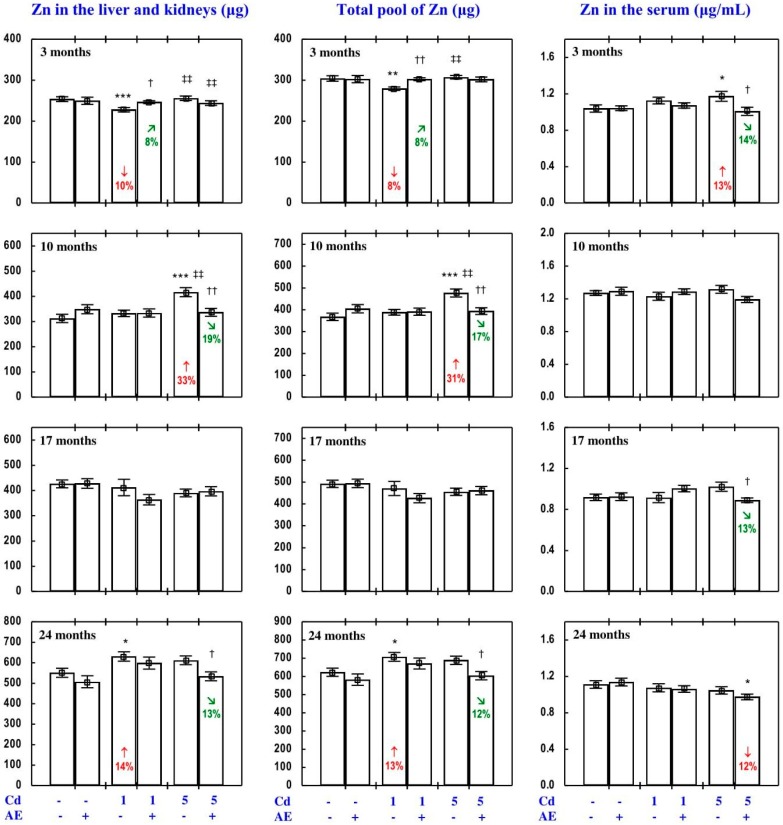
Zinc (Zn) content in the liver and kidneys and its total pool in internal organs, as well as the serum concentration of this element in particular experimental groups. The rats received cadmium (Cd) in their diet at the concentration of 0, 1, and 5 mg/kg and/or 0.1% extract from the berries of *Aronia melanocarpa* (AE; “+”, received; “−”, not received). Data represent mean ± SE for eight rats (except for seven animals in the AE, Cd_1_, and Cd_5_ groups after 24 months). Statistically significant differences (ANOVA, Duncan’s multiple range test): * *p* < 0.05, ** *p* < 0.01, *** *p* < 0.001 vs. control group; ^†^ *p* < 0.05, ^††^ *p* < 0.01 vs. respective group receiving Cd alone; ^‡‡^ *p* < 0.01 vs. respective group receiving the 1 mg Cd/kg diet (alone or with AE) are marked. Numerical values in bars indicate percentage changes compared to the control group (↓, decrease; ↑, increase) or the respective group receiving Cd alone (↘, decrease; ↗, increase).

The administration of AE alone for up to 24 months had no impact on Zn concentration in the serum, soft tissues, and bone tissue (Figure 2, Figure 3, Figure 4, Figure 5 and Figure 6), with a few exceptions (Supplementary Material—Zinc 2).

As is presented in detail in Figure 2, Figure 3, Figure 4, Figure 5 and Figure 6, exposure to Cd alone influenced (increased or decreased) Zn concentration in the serum, soft tissues, and bone tissue depending on the level and duration of treatment. In the rats fed with the 1 mg Cd/kg diet alone, the most important change was an increase in Zn concentration in the liver and kidney with a simultaneous decrease in its concentration in the spleen after 24 months (Figure 2). Exposure to the 5 mg Cd/kg diet alone resulted in considerably varied transitional changes in serum and tissue Zn concentration. The kidney and spleen concentration of this bioelement only increased after 17 months, while its liver concentration was unaffected throughout the study (Figure 2). The bone tissue concentration of this bioelement at both levels of treatment with Cd first decreased after 10 months; however, the change was transitional (Figure 4).

The consumption of AE by the rats fed with diets containing 1 and 5 mg Cd/kg modified the Zn concentration in the serum and tissues (Figure 2, Figure 3, Figure 4, Figure 5 and Figure 6; Supplementary Material—Zinc 3). The administration of the extract to the animals intoxicated with the 1 mg Cd/kg diet entirely prevented Cd-induced changes in liver, kidney, and spleen concentration after 24 months (Figure 2), as well as it prevented a decrease in Zn concentration in the bone tissue at the femoral diaphysis after 10 months of the experiment (Figure 4). The extract consumption under exposure to the 5 mg Cd/kg diet completely prevented a Cd-induced decrease in Zn concentration in the spleen after 3 months (Figure 2). Moreover, the administration of the extract under moderate Cd exposure entirely prevented a Cd-induced increase in Zn concentration in the serum (Figure 6) and its decrease in the femoral muscle and duodenum after 3 months (Figure 4 and Figure 5), in addition to an increase in its concentration in the brain after 10 months (Figure 3). Furthermore, the consumption of the extract for 3 and 10 months partially prevented the decrease in the stomach (Figure 3) and bone tissue (Figure 4) concentration of this bioelement, respectively. As is evident from the data presented in Figure 2, Figure 3, Figure 4 and Figure 5, the administration of AE under moderate exposure to Cd did not protect against all changes in tissue Zn concentrations. The ANOVA/MANOVA analysis (Tables S5 and S6) revealed that the impact of AE administration to the rats fed with the diets containing 1 and 5 mg Cd/kg on Zn concentration in the serum and tissues, including its protective influence against Cd impact on this bioelement concentration, was the result of independent action of the extract ingredients (F = 4.59–15.2, *p* < 0.05–0.001) and/or their interaction with Cd (F = 4.55–34.7, *p* < 0.05–0.001). However, in some cases, the two-way analysis of variance revealed the lack of a statistically significant independent effect of AE and its interaction with Cd (Tables S5 and S6) on serum and tissue Zn concentration, in spite of the evident impact of the extract administration under Cd exposure noticed on the basis of the findings of one-way analysis of variance (Duncan’s multiple range test), presented in Figure 2, Figure 3, Figure 4, Figure 5 and Figure 6.

#### 3.2.3. Zn Content in Internal Organs 

The administration of AE alone for up to 24 months had no impact on Zn content in particular internal organs (liver, kidney, heart, spleen, and brain). The total content of this bioelement in the liver and kidneys, as well as its total pool in internal organs did not change, either, as a result of AE consumption (Figure 6, Figures S2 and S3).

The impact of low and moderate exposure to Cd on the total pool of Zn mainly involved changes in the content of this bioelement in the liver and kidneys (Figure 6 and Figure S2). The 3-month low-level exposure to Cd resulted in a decrease in the total pool of Zn in internal organs (by 8%) connected with a decrease (by 10%) in the total content of this bioelement in the liver and kidneys (resulting from lower Zn content in the liver). The application of the 1 mg Cd/kg diet for 10 and 17 months had no impact on Zn content in particular internal organs and thus its total pool in these organs (Figure 6, Figures S2 and S3). Twenty-four-month intoxication led to an increase in the total pool of Zn in internal organs (by 13%), which was connected with increased (by 14%) total Zn content in the liver and kidneys (determined by a 7% increase in Zn content in the kidneys) and in the heart (determined by a 12% increase; Figure 6 and Figure S2). Ten months of feeding with the 5 mg Cd/kg diet increased (by 31%) the total pool of Zn in internal organs, and the change resulted from its enhanced content in the liver, kidneys, and brain (Figure 6, Figures S2 and S3). The content of Zn in the kidneys also increased after 17 and 24 months of moderate exposure (by 17% and 5%, respectively; Figure S2); however, the total pool of this bioelement in internal organs was unchanged (Figure 6).

The administration of AE under exposure to the 1 and 5 mg Cd/kg diets modified Zn content in particular internal organs and its total pool in these organs (Figure 6, Figures S2 and S3). The extract intake, when treating with the 1 mg Cd/kg diet, completely prevented the abovementioned changes in the total pool of Zn in internal organs and the sum of this bioelement content in the liver and kidneys, as well as in the content of Zn in the liver (after 3 months) and kidney and heart (after 24 months; Figure 6 and Figure S2). The 10-month administration of AE to the animals fed with the diet containing 5 mg Cd/kg entirely prevented the Cd-induced increase in the total pool of Zn in internal organs, the sum of its content in the liver and kidneys, and its content in the liver and brain (Figure 6, Figures S2 and S3). The administration of AE under exposure to the 1 and/or 5 mg Cd/kg diet changed (increased or decreased) the Cd-unaffected content of Zn in some internal organs (liver, heart, spleen, and brain) in comparison to the control/or relevant Cd group (Figure 6, Figures S2 and S3; Supplementary Material—Zinc 3). The ANOVA/MANOVA analysis (Table S7) revealed that the effect of the extract co-administration on the content of Zn in internal organs, including its protective impact against the influence of Cd on this bioelement content in internal organs, was the result of its independent influence (F = 5.00–9.11, *p* < 0.05–0.01) and/or interaction with Cd (F = 5.31–12.6, *p* < 0.05–0.01). However, in some cases the analysis revealed the lack of a statistically significant independent effect of AE and its interaction with Cd (Table S7) on the content of Zn in particular organs and its total pool in internal organs, in spite of the evident impact of the AE consumption under Cd exposure noticed on the basis of the results of one-way analysis of variance (Duncan’s multiple range test) presented in Figure 6, Figures S2 and S3.

### 3.3. Effect of AE on the Body Status of Cu under Exposure to Cd

#### 3.3.1. Cu Apparent Absorption, Retention in the Body, and Its Faecal and Urinary Excretion

**Figure 7:**
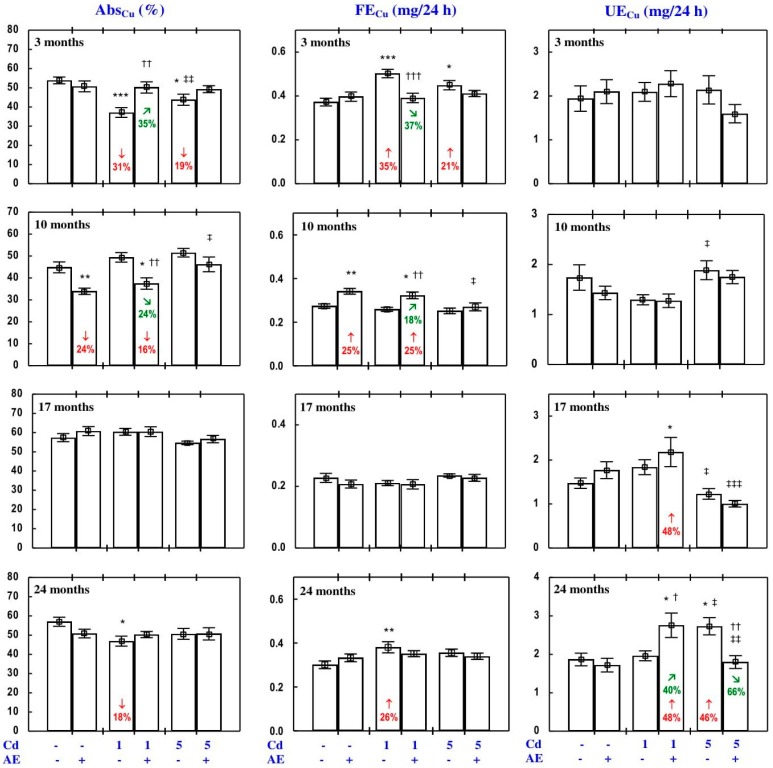
The apparent absorption of copper (Abs_Cu_) and its daily faecal (FE_Cu_) and urinary excretion (UE_Cu_) in particular experimental groups. The rats received cadmium (Cd) in their diet at the concentration of 0, 1, and 5 mg/kg and/or 0.1% extract from the berries of *Aronia melanocarpa* (AE; “+”, received; “-”, not received). Data represent mean ± SE for eight rats (except for seven animals in the AE, Cd_1_, and Cd_5_ groups after 24 months). Statistically significant differences (ANOVA, Duncan’s multiple range test): * *p* < 0.05, ** *p* < 0.01, *** *p* < 0.001 vs. control group; ^†^ *p* < 0.05, ^††^ *p* < 0.01, ^†††^ *p* < 0.001 vs. respective group receiving Cd alone; ^‡^ *p* < 0.05, ^‡‡^ *p* < 0.01, ^‡‡‡^ *p* < 0.001 vs. respective group receiving the 1 mg Cd/kg diet (alone or with AE) are marked. Numerical values in bars indicate percentage changes compared to the control group (↓, decrease; ↑, increase) or the respective group receiving Cd alone (↘, decrease; ↗, increase).

The apparent absorption and body retention of Cu (Abs_Cu_ and Ret_Cu_, respectively) in the control animals reached 44–57% (Figure 7 and Figure S1). The administration of AE alone had no impact on the Abs_Cu_ and Ret_Cu_ or urinary and faecal excretion of this bioelement (UE_Cu_ and FE_Cu_, respectively; Figure 7 and Figure S1), with some exceptions (Supplementary Material—Copper 1).

In the rats exposed to the 1 mg Cd/kg diet alone for 3 and 24 months, the Abs_Cu_ and Ret_Cu_ were lower, while the FE_Cu_ was higher compared to the control group (Figure 7 and Figure S1). Three months of exposure to the 5 mg Cd/kg diet decreased the Abs_Cu_ and Ret_Cu_, and increased the FE_Cu_ (Figure 7 and Figure S1). Low-level exposure to Cd had no impact on the UE_Cu_, whereas after the 24-month moderate exposure the value of this parameter increased (Figure 7). The consumption of AE by the animals fed with the diets containing Cd modified its influence on the Abs_Cu_, Ret_Cu_, FE_Cu_, and UE_Cu_ (Figure 7 and Figure S1). The administration of AE completely prevented the decrease in the Abs_Cu_ and Ret_Cu_ as well as the increase in the FE_Cu_ caused by 3 and 24 months of exposure to the 1 mg Cd/kg diet (Figure 7 and Figure S1). The consumption of AE by the rats fed with the 5 mg Cd/kg diet completely prevented all Cd-caused changes in the Abs_Cu_, Ret_Cu_, FE_Cu_, and UE_Cu_ (Figure 7 and Figure S1). The ANOVA/MANOVA analysis (Table S8) revealed that the influence of co-administration of the extract and Cd on the Abs_Cu_, Ret_Cu_, FE_Cu_, and UE_Cu_, including its protection against the unfavourable effects of Cd, was the result of independent action of the extract (F = 4.72–29.0, *p* < 0.05–0.001) and/or interaction of its ingredients with the toxic metal (F = 4.57–14.8, *p* < 0.05–0.001). However, at some time points, two-way analysis of variance revealed the lack of a statistically significant independent effect of AE and its interaction with Cd (Table S8) on the Abs_Cu_, Ret_Cu_, FE_Cu_, and UE_Cu_, in spite of the evident impact of administration of the extract under Cd exposure observed on the basis of the findings of one-way analysis of variance (Duncan’s multiple range test), presented in Figure 7 and Figure S1.

#### 3.3.2. Cu Concentration in the Serum and Tissues 

**Figure 8:**
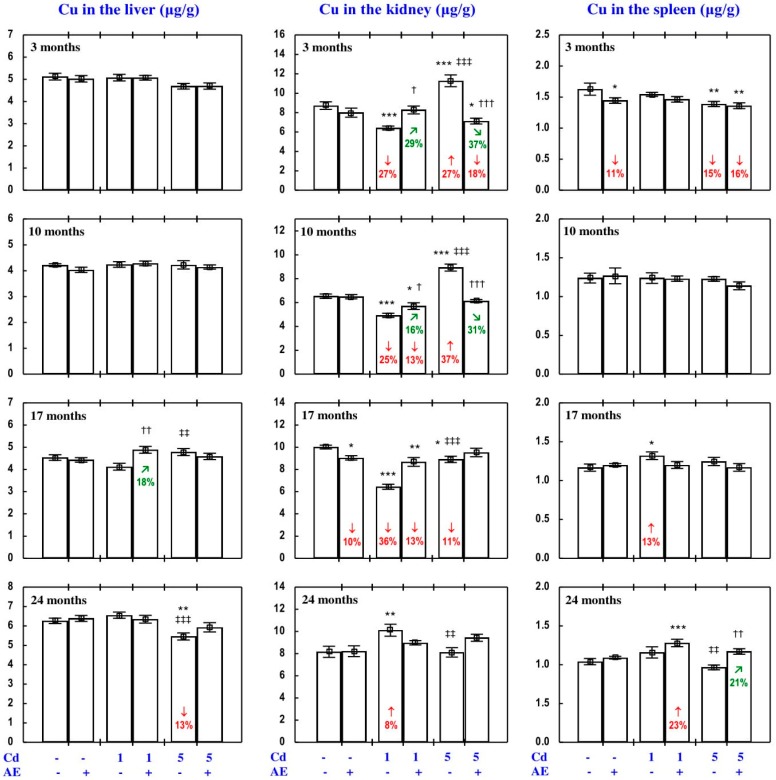
Copper (Cu) concentration in the liver, kidney, and spleen in particular experimental groups. The rats received cadmium (Cd) in their diet at the concentration of 0, 1, and 5 mg/kg and/or 0.1% extract from the berries of *Aronia melanocarpa* (AE; “+”, received; “-”, not received). Data represent mean ± SE for eight rats (except for seven animals in the AE, Cd_1_, and Cd_5_ groups after 24 months). Statistically significant differences (ANOVA, Duncan’s multiple range test): * *p* < 0.05, ** *p* < 0.01, *** *p* < 0.001 vs. control group; ^†^ *p* < 0.05, ^††^ *p* < 0.01, ^†††^ *p* < 0.001 vs. respective group receiving Cd alone; ^‡‡^ *p* < 0.01, ^‡‡‡^ *p* < 0.001 vs. respective group receiving the 1 mg Cd/kg diet (alone or with AE) are marked. Numerical values in bars indicate percentage changes or factors of changes compared to the control group (↓, decrease; ↑, increase) or the respective group receiving Cd alone (↘, decrease; ↗, increase).

**Figure 9:**
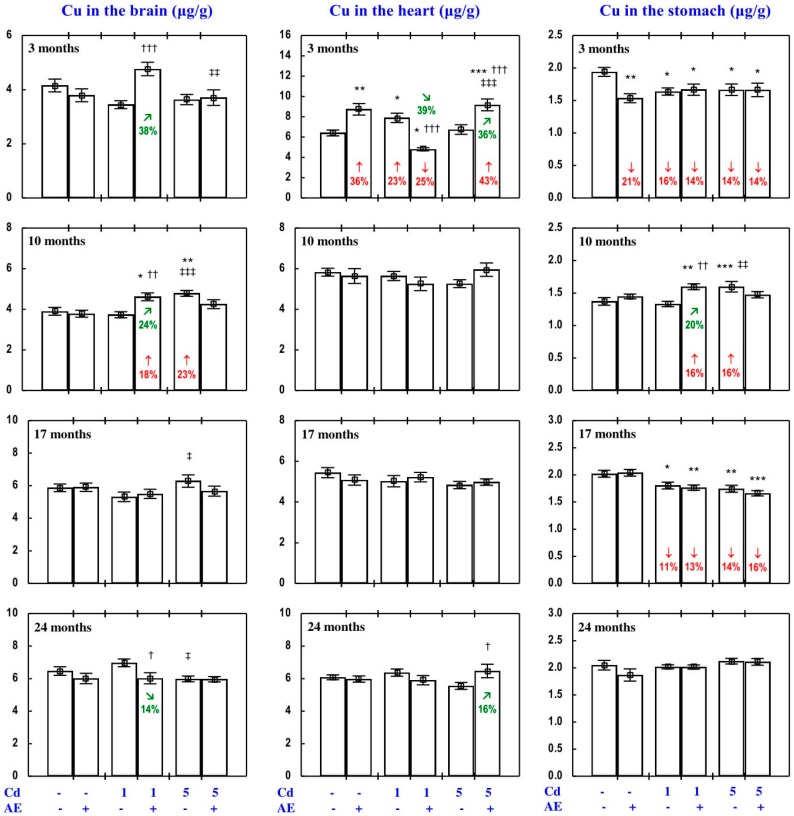
Copper (Cu) concentration in the brain, heart, and stomach in particular experimental groups. The rats received cadmium (Cd) in their diet at the concentration of 0, 1, and 5 mg/kg and/or 0.1% extract from the berries of *Aronia melanocarpa* (AE; “+”, received; “-”, not received). Data represent mean ± SE for eight rats (except for seven animals in the AE, Cd_1_, and Cd_5_ groups after 24 months). Statistically significant differences (ANOVA, Duncan’s multiple range test): * *p* < 0.05, ** *p* < 0.01, *** *p* < 0.001 vs. control group; ^†^ *p* < 0.05, ^††^ *p* < 0.01, ^†††^ *p* < 0.001 vs. respective group receiving Cd alone; ^‡^ *p* < 0.05, ^‡‡^ *p* < 0.01, ^‡‡‡^ *p* < 0.001 vs. respective group receiving the 1 mg Cd/kg diet (alone or with AE) are marked. Numerical values in bars (or above the bars) indicate percentage changes compared to the control group (↓, decrease; ↑, increase) or the respective group receiving Cd alone (↘, decrease; ↗, increase).

**Figure 10:**
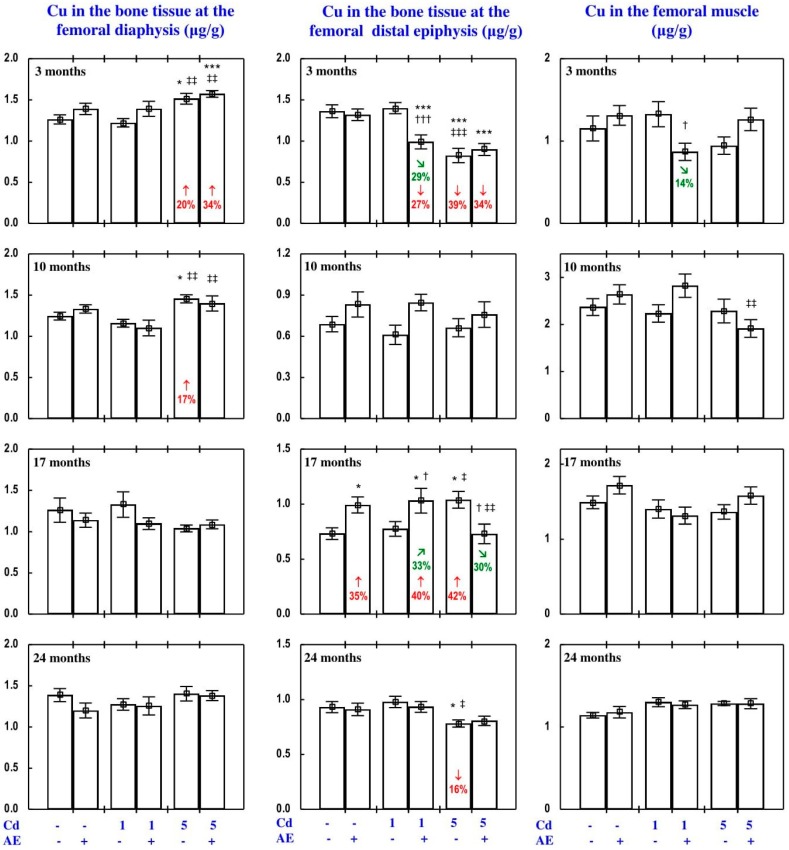
Copper (Cu) concentration in the bone tissue and femoral muscle in particular experimental groups. The rats received cadmium (Cd) in their diet at the concentration of 0, 1, and 5 mg/kg and/or 0.1% extract from the berries of *Aronia melanocarpa* (AE; “+”, received; “-”, not received). Data represent mean ± SE for eight rats (except for seven animals in the AE, Cd_1_ and Cd_5_ groups after 24 months). Statistically significant differences (ANOVA, Duncan’s multiple range test): * *p* < 0.05, *** *p* < 0.001 vs. control group; ^†^ *p* < 0.05, ^†††^ *p* < 0.001 vs. respective group receiving Cd alone; ^‡^ *p* < 0.05, ^‡‡^ *p* < 0.01, ^‡‡‡^ *p* < 0.001 vs. respective group receiving the 1 mg Cd/kg diet (alone or with AE) are marked. Numerical values in bars indicate percentage changes compared to the control group (↓, decrease; ↑, increase) or the respective group receiving Cd alone (↘, decrease; ↗, increase).

**Figure 11:**
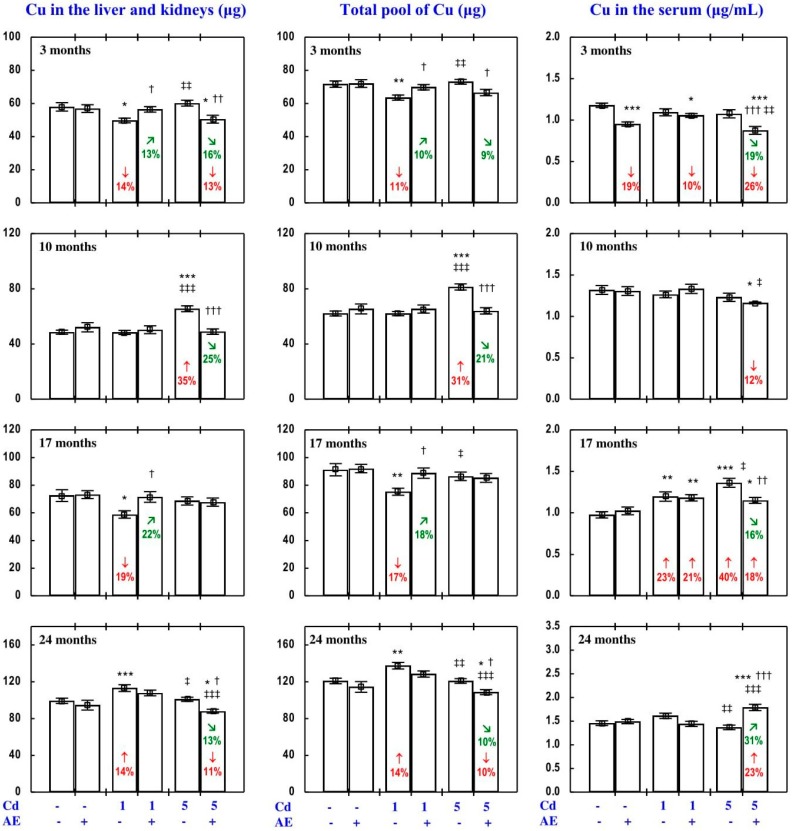
Copper (Cu) content in the liver and kidneys and its total pool in internal organs, as well as the serum concentration of this element in particular experimental groups. The rats received cadmium (Cd) in their diet at the concentration of 0, 1, and 5 mg/kg and/or 0.1% extract from the berries of *Aronia melanocarpa* (AE; “+”, received; “-”, not received). Data represent mean ± SE for eight rats (except for seven animals in the AE, Cd_1_ and Cd_5_ groups after 24 months). Statistically significant differences (ANOVA, Duncan’s multiple range test): * *p* < 0.05, ** *p* < 0.01, *** *p* < 0.001 vs. control group; ^†^ *p* < 0.05, ^††^ *p* < 0.01, ^†††^ p < 0.001 vs. respective group receiving Cd alone; ^‡^ *p* < 0.05, ^‡‡^ *p* < 0.01, ^‡‡‡^ *p* < 0.001 vs. respective group receiving the 1 mg Cd/kg diet (alone or with AE) are marked. Numerical values in bars indicate percentage changes compared to the control group (↓, decrease; ↑, increase) or the respective group receiving Cd alone (↘, decrease; ↗, increase).

The administration of AE alone for up to 24 months had no impact on Cu concentration in the serum, soft tissues, and bone tissue (Figure 5, Figure 8, Figure 9, Figure 10 and Figure 11), with a few exceptions (Supplementary Material—Copper 2).

As is presented in detail in Figure 5, Figure 8, Figure 9, Figure 10 and Figure 11, exposure to the 1 and 5 mg Cd/kg diets alone influenced (increased or decreased) Cu concentration in the serum, soft tissues, and bone tissue depending on the level of the treatment and its duration (Figure 5, Figure 8, Figure 9, Figure 10 and Figure 11). In the rats fed with the 1 mg Cd/kg diet alone, the most important change was a decrease in the kidney concentration of Cu after 3, 10, and 17 months, and its increase after 24 months (Figure 8). Exposure to the 5 mg Cd/kg diet alone resulted in very varied transitional changes in tissue Cu concentrations (Figure 5, Figure 8, Figure 9, Figure 10 and Figure 11). Three months of moderate exposure to Cd decreased and increased the concentration of this bioelement in the spleen and kidney, respectively (Figure 8). Moreover, the kidney concentration of Cu increased after 10 months of the treatment and decreased after 17 months (Figure 8). Exposure to the 5 mg Cd/kg diet alone only decreased Cu concentration in the liver after 24 months (Figure 8). Pronounced changes of Cu concentration were also evident in the bone tissue; they depended on the duration of exposure and the kind of the bone tissue (Figure 10). Moderate Cd treatment increased Cu concentration in the bone tissue at the femoral diaphysis after 3 and 10 months, while its concentration in the bone tissue at the femoral distal epiphysis decreased after 3 and 24 months and increased after 17 months (Figure 10).

The administration of AE under exposure to the 1 and 5 mg Cd/kg diets modified Cu concentration in the serum and tissues (Figure 5, Figure 8, Figure 9, Figure 10 and Figure 11). The administration of the extract to the rats fed with the 1 mg Cd/kg diet for 3 and 10 months prevented (entirely and partially, respectively) against the Cd-induced decrease in kidney Cu concentration, but it had no protective impact against its decrease after 17 months (however, Cu concentration in the Cd_1_ + AE group was lower by 13% than that in the control group, whereas in the Cd_1_ group it decreased by 36%), and after 24 months it completely prevented the Cd-caused increase in the concentration of this bioelement in the kidney and duodenum (Figure 5 and Figure 8). AE administration during the 3 months of exposure to the 1 mg Cd/kg diet decreased the Cd-enhanced Cu concentration in the heart compared to the control group and Cd_1_ group (Figure 9). The administration of AE under the treatment with the 5 mg Cd/kg diet completely prevented the Cd-induced increase in Cu concentration in the kidney after 3 and 10 months as well as the decrease in this bioelement concentration in the kidney after 17 months; however, the kidney Cu concentration in the Cd_5_ + AE group after 3 months was lower than that in the control group (Figure 8). Moreover, the intake of the extract under the moderate Cd treatment completely prevented the Cd-induced increase in Cu concentration in the brain and stomach after 10 months (Figure 9), and in bone tissue at the femoral diaphysis and distal epiphysis after 10 and 17 months, respectively (Figure 10), as well as the decrease in Cu concentration in the liver (Figure 8) and bone tissue at the femoral distal epiphysis caused by 24 months of exposure (Figure 10). As is evident from the data presented in Figure 5, Figure 8, Figure 9, Figure 10 and Figure 11, the administration of AE under the moderate exposure to Cd did not protect against all changes in Cu concentrations, but the concentration of this bioelement was modified (decreased or increased) in the serum and various tissues that were unchanged by this heavy metal (Supplementary Material—Copper 2). According to the results of the ANOVA/MANOVA analysis (Tables S9 and S10), the modifying impact of AE consumption on Cu concentration in the serum and tissues under the low-level and moderate exposure to Cd was the result of independent action of the extract (F = 4.62–48.3, *p* < 0.05–0.001) and/or interaction of its ingredients with this toxic metal (F = 5.28–46.0, *p* < 0.05–0.001). However, at some time points, the two-way analysis of variance revealed the lack of a statistically significant independent effect of AE and its interaction with Cd (Tables S9 and S10) on serum and tissue Cu concentrations, in spite of the evident impact of the administration of the extract under Cd treatment observed on the basis of the findings of one-way analysis of variance (Duncan’s multiple range test), presented in Figure 5, Figure 8, Figure 9, Figure 10 and Figure 11.

#### 3.3.3. Cu Content in Internal Organs 

The administration of AE alone for up to 24 months had no impact on Cu content in particular internal organs (liver, kidney, heart, spleen, brain), nor on the total content of this bioelement in the liver and kidneys, nor its total pool in internal organs (Figure 11, Figures S4 and S5).

The impact of low and moderate exposure to Cd on the total pool of Cu mainly involved changes in the content of this bioelement in the liver and kidneys (Figure 11 and Figure S4). Low-level exposure to Cd decreased the total pool of Cu in internal organs after 3 and 17 months (Figure 11), which resulted from a decrease in its content in the kidney (by 29% and 34%, respectively) and thus also the sum of its content in the liver and kidneys (Figure 11 and Figure S4). Twenty-four months of feeding with the 1 mg Cd/kg diet increased the total pool of this bioelement in internal organs, which was connected with increased Cu content in the liver and kidneys in total, as well as in the heart (Figure 11 and Figure S4). Moreover, low-level exposure to Cd decreased (by 44%) the kidney content of Cu after 10 months, but in spite of that its total pool in internal organs was unchanged (Figure 11 and Figure S4). Moderate exposure to Cd for 3 months increased Cu content in the kidney (by 27%), but this change had no impact on the total pool of this bioelement in internal organs. Ten months of exposure to the 5 mg Cd/kg diet resulted in an increase in Cu content in the liver, kidney, and brain, as well as the sum of its content in the liver and kidneys and the total pool of this bioelement in internal organs (Figure 11 and Figure S4). Seventeen and 24 months of moderate treatment with Cd had no influence on the content of Cu in any of the estimated internal organs, nor on the total content of this bioelement in the liver and kidneys, nor its total pool in internal organs (Figure 11, Figures S4 and S5).

The administration of AE during the exposure to the 1 mg Cd/kg diet entirely prevented the decrease in the sum of Cu content in the liver and kidneys and the total pool of this bioelement in internal organs after 3 and 17 months induced by this toxic metal, as well as the increase in these contents after 24 months (Figure 11). The extract consumption completely prevented the decrease in Cu content in the kidney caused by 3 and 10 months of low-level exposure to Cd, as well as the increase in heart Cu content after 24 months (Figure S4). The administration of AE accompanying feeding with the diet containing 5 mg Cd/kg completely prevented this toxic metal-induced increase in the content of Cu in the liver and kidney alone, the total content of Cu in both organs together, and its total pool in internal organs after 10 months. Moreover, the extract consumption changed (increased or decreased) the content of Cu in some internal organs that were unaffected by Cd (Supplementary Material—Copper 3). The ANOVA/MANOVA analysis (Table S11) revealed that the above influence of *Aronia* extract administration under the low-level and moderate treatment with Cd on the content of Cu in internal organs was the result of independent action of the extract (F = 4.34–49.1, *p* < 0.05–0.001) and/or interaction of its ingredients with this xenobiotic (F = 6.77–54.1, *p* < 0.05–0.001). However, in some cases, two-way analysis of variance revealed the lack of a statistically significant independent effect of AE and its interaction with Cd (Table S11) on Cu content in organs, in spite of the clear impact of the extract administration under Cd treatment recognised on the basis of the findings of one-way analysis of variance (Duncan’s multiple range test) presented in Figure 11, Figures S4 and S5.

### 3.4. Effect of AE on MT Concentration in the Liver, Kidney, and Duodenum, and the Degree of Zn, Cu, and Cd Binding to This Protein

#### 3.4.1. MT Concentration

**Figure 12:**
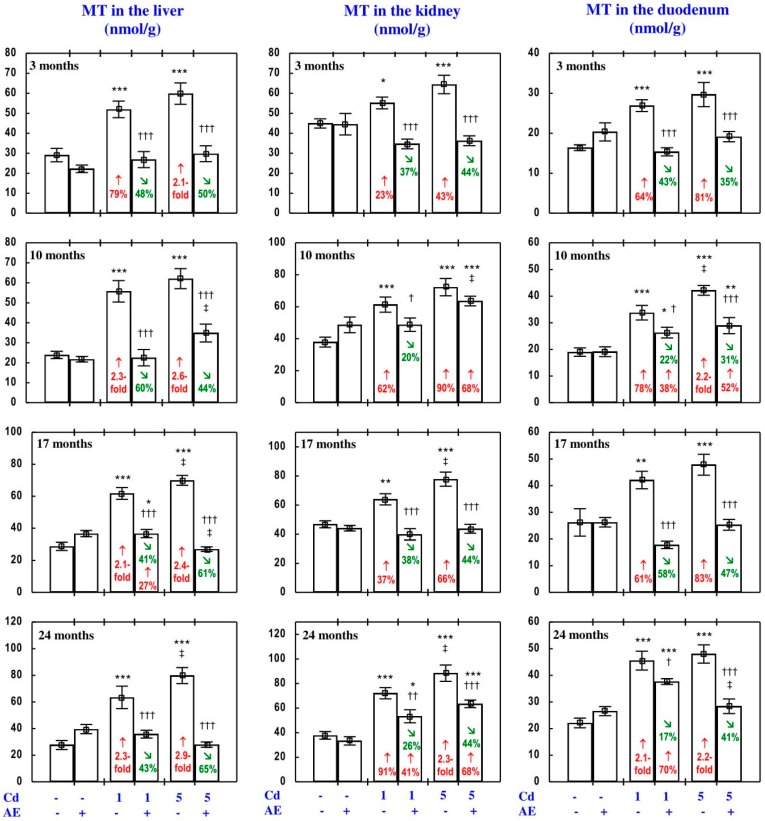
Metallothionein (MT) concentration in the liver, kidney, and duodenum in particular experimental groups. The rats received cadmium (Cd) in their diet at the concentration of 0, 1, and 5 mg/kg and/or 0.1% extract from the berries of *Aronia melanocarpa* (AE; “+”, received; “-”, not received). Data represent mean ± SE for eight rats (except for seven animals in the AE, Cd_1_, and Cd_5_ groups after 24 months). Statistically significant differences (ANOVA, Duncan’s multiple range test): * *p* < 0.05, ** *p* < 0.01, *** *p* < 0.001 vs. control group; ^†^ *p* < 0.05, ^††^ *p* < 0.01, ^†††^ *p* < 0.001 vs. respective group receiving Cd alone; ^‡^ *p* < 0.05 vs. respective group receiving the 1 mg Cd/kg diet (alone or with AE) are marked. Numerical values in bars indicate percentage changes or factors of changes compared to the control group (↑, increase) or the respective group receiving Cd alone (↘, decrease).

The administration of AE alone for up to 24 months had no impact on MT concentration in the liver, kidney, and duodenal tissue (Figure 12).

In the rats fed with the diet containing 1 and 5 mg Cd/kg, the concentration of MT in the liver, kidney, and duodenum at all evaluated time points was markedly higher (from 23% to 2.9-fold) compared to the control group (Figure 12). MT concentration in the liver and kidney was higher than that in the duodenal tissue.

In the animals receiving AE alongside treatment with the 1 and 5 mg Cd/kg diets, the concentration of MT in the liver, kidney, and duodenum at each time point was markedly lower compared to the respective group exposed to Cd alone, and in some of the cases it did not differ from the control group, except for the lack of influence of the extract on this protein’s concentration in the kidney in the Cd_5_ + AE group after 10 months of the experiment (Figure 12). This indicates that the extract consumption completely or partially prevented the heavy metal-induced increase in the concentration of MT in these tissues. According to the results of ANOVA/MANOVA analysis (Tables S12–S14), the impact of AE on MT concentration in the liver, kidney, and duodenum described above was the result of independent action of the extract (F = 7.2–54.8, *p* < 0.05–0.001) and/or interaction of its ingredients with Cd (F = 4.86–115.2, *p* < 0.05–0.001), except for the protein’s concentration in the duodenum in the Cd_1_ + AE group after 10 months of the experiment. Although MT concentration in the duodenal tissue in this group was lower compared to the Cd_1_ group and higher than that in the control group (Figure 12), the ANOVA/MANOVA revealed that neither AE alone nor its interaction with Cd had a significant effect on this protein’s concentration (Table S14).

#### 3.4.2. The Degree of Zn, Cu, and Cd Binding to MT 

The administration of AE alone decreased the pool of non-MT-bound Zn and Cu (Zn/(MT × 7) and Cu/(MT × 12), respectively) and the total pool of MT-unbound Zn, Cu, and Cd (Me/Me-MT) in the liver after 17 and 24 months (Table 3) and in the kidney after 10 months (Table 4), as well as Me/Me-MT in the duodenum after 10 months and the duodenal ratios of Zn/(MT × 7) and Cu/(MT × 12) after 17 and 24 months, compared to the control group (Table 5). The administration of the extract alone throughout the whole experimental period had no impact on MT saturation with Cd (Cd/(MT × 7); Table 3, Table 4 and Table 5).

Feeding of the rats with the 1 and 5 mg Cd/kg diets changed the degree of Zn, Cu, and Cd binding to MT, as well as the total pool of MT-unbound metals in the liver, kidney, and duodenum in different ways, depending on the level and duration of the treatment (Table 3, Table 4 and Table 5). Generally, low and moderate exposure to Cd increased the liver, kidney, and duodenal MT saturation with this toxic metal, except for a lack of impact on the pool of non-MT bound Cd in the liver in the case of the low-level treatment (Table 3, Table 4 and Table 5). Moreover, as is evident from the detailed data presented in Table 3, Table 4 and Table 5, exposure to both levels of Cd alone decreased the pool of MT-unbound Zn and Cu, as well as the total pool of MT-unbound metals in the liver, kidney, and duodenum, with only a few exceptions (Table 3, Table 4 and Table 5).

The administration of AE to the animals fed with the diets containing 1 and 5 mg Cd/kg either had no impact or increased the pool of non-MT-bound Cd in the liver, kidney, and duodenum (Table 3, Table 4 and Table 5). Moreover, the intake of AE under exposure to Cd significantly (partially or completely), prevented the decrease in the pool of MT-unbound Zn and Cu, as well as in the total pool of metals unbound to this protein, induced by Cd (Table 3, Table 4 and Table 5). The ANOVA/MANOVA analysis (Tables S12–S14) revealed that the modifying impact of AE consumption while feeding with the diets containing 1 and 5 mg Cd/kg on the degree of Zn, Cu, and Cd binding to the liver, kidney, and duodenal MT, as well as on the total pool of MT-unbound metals, was the result of independent action of the extract ingredients (F = 4.22–79.3, *p* < 0.05–0.001) and/or their interaction with this toxic metal (F = 4.49–80.1, *p* < 0.05–0.001).

## 4. Discussion

The present study is the first to investigate and reveal the modifying effect of prolonged consumption of a polyphenol-rich AE on the body status of Zn and Cu under chronic low-level and moderate exposure to Cd via diet. Although the study was focused first of all on the influence of AE on the metabolism of both bioelements in the conditions of chronic intoxication with this toxic metal, it has also provided new important data on their body status in the case of chokeberry extract consumption under the standard diet, and on the influence of low-level and moderate lifetime exposure to Cd on the metabolism of these necessary elements. It has been reported that repeated excessive exposure to Cd results in the redistribution of Zn and Cu in the organism, mainly consisting in the retention of these bioelements in the liver and kidneys in the MT-bound form and a decrease in their concentrations in the serum and other organs and tissues, including bone tissue [15,22,36,38,52,53,54,55]. However, there is no complex evaluation of the influence of long-term low-level exposure on the body status of Zn and Cu, properly reflecting the environmental exposure to Cd occurring in industrialised countries nowadays [14,15,16,17,18,19,20,21,48,49].

The relevance of the used experimental model for investigating the impact of Cd or/and AE on the body status of Zn and Cu is confirmed by the same daily intake of particular bioelements in all groups throughout the experiment, as well as the same intake of Cd at particular levels of exposure regardless of whether this xenobiotic was administered alone or in conjunction with AE, and the same consumption of AE in the groups receiving the extract alone and together with Cd. The fact that the daily intake of Zn and Cu in all of the groups during the first 3 months of the study was higher than that during the other experimental periods resulted from higher Zn and Cu content in the breeding diet administered during the first 3 months, compared to the maintenance diet used thereafter, as well as from lower body mass of the young animals compared to the older ones, with similar food consumption [7].

The present study has revealed that treatment with Cd, reflecting moderate human exposure to this heavy metal (5 mg Cd/kg diet) and corresponding to general population’s the low-level exposure in industrialised countries (1 mg Cd/kg diet), may cause disorders in the metabolism of Zn and Cu. Percentage changes in the values of estimated indices of the body status of Zn and Cu induced by the 1 mg Cd/kg diet were relatively low. However, the fact that they occurred at such low exposure levels makes them very important, as Cd is a common and unavoidable pollutant of food products [13]. The decreased total pool of Zn in internal organs and the sum of this bioelement content in the liver and kidneys as a result of 3 months of administration of the diet containing 1 mg Cd/kg, despite the lack of statistically significant changes of its concentration and content in particular internal organs or in the serum and bone tissue concentrations, indicate a slight deficiency of this bioelement in the organism, associated with its deficiency in the liver and kidneys. The decreased total pool of Zn in internal organs after 3 months of feeding with the 1 mg Cd/kg diet, together with its unaffected total pool after 10 and 17 months, and the fact that dietary intake of this bioelement during the first 3 months of the study was higher than thereafter, suggest that a young organism may be particularly susceptible to Zn deficiency even in the case of low-level exposure to Cd. The slight decrease in the content of Zn in internal organs of the rats fed with the 1 mg Cd/kg diet for 3 months, including the sum of its content in the liver and kidneys (being the main organs of this bioelement storage in the body), may be explained by interactions at the stage of gastrointestinal absorption and transport of both metals in the organism [33,56]. This is confirmed by the decreased Zn concentration in the duodenal tissue and stomach of these animals, as well as being consistent with the available literature data on Cd–Zn interactions, including our own findings [15,22,23,24,25,26,27,33,34,35,36,37,38,39,45,47,52,53,54]. Interactions between Cd and Zn at the absorption stage from the digestive tract are related, first of all, to metals’ ability to both synthesise MT in the duodenum and bind to it [33]. Moreover, these elements may compete for uptake by this protein and zinc-regulated transporter proteins [33,56]. The unchanged value of the Abs_Zn_ in the Cd_1_ group after 3 months, reflecting Zn bioavailability from the digestive tract (estimated based on the difference between its daily oral intake and faecal excretion), does not rule out a slight decrease in its absorption. The decreased value of the Abs_Zn_ in this experimental group after 10 months confirms the capability of Cd to decrease Zn absorption from the gastrointestinal tract in the case of low-level exposure.

The next very important finding is that low-level lifetime (24 months in our experimental model) exposure to this xenobiotic, unlike exposure in youth only (3 months in our experimental model), may result in the enhanced retention of Zn in the liver and kidneys, and thus also enhance its total pool in internal organs (in spite of its decreased content in the spleen). This is in good agreement with the literature data on this bioelement accumulation in the liver and kidneys under environmental exposure to Cd [15,45,52] and intoxication with this heavy metal in animal models [22,37,45]. The detailed mechanism of Zn retention in the liver and kidneys, which is connected with Cd accumulation and MT biosynthesis in these organs, is widely reported [22,33,37,38]. An increase in the concentrations of Cd and MT in the liver and kidneys may be accompanied with a simultaneous increase in the content of Zn and Cu [22,37], as was observed in the present study in the rats fed for 24 months with the diet containing 1 mg Cd/kg.

It should be emphasised that the lifetime moderate treatment with Cd, unlike the low-level exposure, did not result in Zn retention in the liver and kidneys in spite of the slightly increased (by 5%) concentration of this bioelement in the kidney, as well as Cd accumulation in the liver and kidney [7] and markedly enhanced MT concentration in these organs. However, the higher total pool of Zn in internal organs, as well as its content in the liver and kidney after 10 months of exposure to the 5 mg Cd/kg diet together with the increased Zn content in the kidney after 17 months, show that under moderate exposure to Cd, a rise in the content of Zn in the liver and kidneys did occur as well, but due to higher Cd [7] and MT concentrations in these organs it took place after a shorter period of exposure than in the case of low-level treatment, and it was temporary. The fact that 24 months of moderate exposure to Cd resulted in an unchanged total pool of Zn in internal organs and its content in the liver and kidneys after their previous rise allow us to suppose that in the last phase of lifetime moderate exposure to Cd, interactions between Cd and Zn, including those in the gastrointestinal tract resulting in a decrease in Zn absorption, might occur. Because of the accumulation of Zn in internal organs during the earlier stage of exposure, the possibly decreased intake of this bioelement at the further stage might ensure that the body content of this bioelement is kept at the correct level, as was reflected in the proper concentration and content of Zn in tissues and organs after 24 months. Moreover, detailed analysis of the results on the body status of Zn in the females fed with the 5 mg Cd/kg diet allowed us to conclude that the increased value of the Abs_Zn_ noted after 24 months of the experiment might result from the enhanced retention of this bioelement in the gastrointestinal tract or might be a compensatory mechanism preventing Zn deficiency in the organism.

The results of the present study show that in the case of low-level exposure to Cd, a young organism is also susceptible to Cu deficiency, like in the case of Zn, while lifetime intoxication with this toxic metal also results in an increase in the total pool of this bioelement in internal organs. Both the deficiency of Cu at a young age and its increased total pool in internal organs due to lifetime low-level exposure were associated with a change of its concentration and content in the kidneys. The decreased Abs_Cu_ and Ret_Cu_ in the young animals fed for 3 months with the diet containing 1 mg Cd/kg confirm that decreased gastrointestinal absorption of Cu and its lower retention in the body were the cause of the lower pool of this element in internal organs, mostly determined by its decreased content in the kidneys. The clear tendency to increase the Cu content in the kidney of the animals treated with the 1 mg Cd/kg diet for 24 months, together with the higher sum of its content in the liver and kidneys in spite of the unchanged concentration and content of this element in the liver, show that the increase in the total pool of Cu in internal organs of these animals is connected with its accumulation in the kidney. The possible mechanism explaining the changes of the body status of Cu seems to be analogous to the case of Zn; however, under lifetime low-level exposure, Cu was mainly retained in the kidneys, while Zn was mainly retained in the liver and kidneys.

The findings of the present study on the impact of 24 months of administration of a diet containing 1 mg Cd/kg on the liver and kidney status of Zn and Cu are in agreement with the results obtained by Noël and others [47] in female rats exposed for 13 weeks to a 10 mg Cd/kg diet and by Piasek and others [53] in pregnant rats intoxicated via diet with 3 and 5 mg Cd/kg b.w. Moreover, they are consistent with our previous results in male rats treated with 50 mg Cd/L in drinking water for 12 weeks [38] and 6 months [22].

Based on the results of the present study, it can be concluded that under low-level and moderate chronic exposure to Cd, changes may occur in the bone concentrations of bioelements necessary for the proper metabolism of the bone tissue, such as Zn and Cu [57], and the changes depend on the level and duration of exposure on the one hand and the kind of bone tissue on the other hand. Generally, the changes noticed in the bone concentrations of Zn and Cu consisted in their deficiency (with only a few exceptions). The decrease in the bone concentrations of Zn and Cu may be explained by Cd-induced disorders in the bone tissue metabolism (inhibition of bone formation and stimulation of bone resorption) noted by us in the females maintained on the 1 and 5 mg Cd/kg diets [5,9], and it may be related to the impact of this xenobiotic on the body status of these bioelements. However, based on our knowledge, we are unable to explain the proper reason for the increase in the bone concentration of Cu. Detailed analysis of the results on the impact of Cd on the bone tissue concentrations of Zn and Cu shows that the low-level lifetime exposure to this heavy metal may only cause a slight and transient decrease in Zn concentration in the regions of the skeleton where compact bone predominates and may have no impact on the concentration of Cu in both trabecular and compact bone regions of the skeleton, while moderate exposure may affect the concentrations of Zn and Cu in both kinds of bone tissue. However, the impact may also be transient and, after a very long period of exposure (24 months), only the concentration of Cu in the regions of the skeleton abundant in the trabecular bone tissue may decrease. The decrease in the bone tissue concentrations of Zn and Cu is in agreement with our previous findings [54,55]; however, this change at such low exposure has not been reported until now.

Our interest in the present study was focused first of all on the impact of prolonged (from 3 up to 24 months) consumption of AE on the body status of Zn and Cu under chronic low-level and moderate exposure to Cd. Moreover, the study allowed us to evaluate the effect of the extract intake on the metabolism of these necessary elements at a very low-level exposure to this toxic metal resulting from its unavoidable trace presence in the standard diet (0.06 mg/kg in our experimental model [7]).

A very important question related to the enhanced consumption of polyphenol-rich products is the risk of decreased bioavailability of divalent bieoelements due to polyphenolic compounds’ ability to bind these elements and form stable, unabsorbable complexes [28,31,32,43,46]. Detailed analysis of the effects on the body status of Zn and Cu in the rats fed with the diet without Cd addition (the AE group) has revealed that long-term and even lifetime (24 months) consumption of AE in the daily dose from 31.1 to 154.7 mg/kg b.w. (corresponding to polyphenols consumption in the dose of 41.5–101.7 mg/kg b.w.) did not disturb the body metabolism of both bioelements in spite of the high content of polyphenols (cyanidin derivatives) and other compounds (fibers and pectins) capable of binding Zn and Cu ions (Zn^2+^ and Cu^2+^) in *Aronia* berries [1,6,28,31,32]. Although the extract intake modified (increased or decreased compared to the proper values of the control group) the concentrations and contents of Zn and Cu in some tissues and organs at some time points, the total pool of these elements in internal organs and their concentrations in the serum and bone tissue were unaffected throughout the 3- to 24-month consumption of the extract, with only a few exceptions. The decrease in the serum Cu concentration that occurred in the young animals was transient, but it may suggest that we have to be careful in the case of prolonged administration of *Aronia* products to young individuals. The declined values of the Abs_Zn_ and Abs_Cu_ due to AE administration show that active compounds present in AE may be able to interact with these bioelements in the lumen of the gastrointestinal tract. However, the lack of evidence of Zn and Cu deficiencies after 3 months of AE administration, except for the decreased Cu concentration in the serum, shows that the decrease in the absorption of these bioelements was not long-lasting. Moreover, the Zn and Cu contents in the diet administered to the animals during the first 3 months of the experiment, higher than thereafter, might counteract the possible negative effect of AE on the gastrointestinal absorption of these bioelements. The decreased values of the Abs_Zn_ and Abs_Cu_, noted after 10 months of AE consumption, may be explained by Zn and Cu binding by some ingredients of the extract [1,6,28,31,32]; however, these had no influence on the body status of both bioelements. The finding that even long-term consumption of AE together with the diet containing trace amounts of Cd has no negative influence on the body status of Zn and Cu is a very important result of this study. It indicates that rational amounts of *A. melanocarpa* berries containing-products may be included in the daily diet without the risk of disturbances in the metabolism of these elements. This finding is of practical use because the consumption of *Aronia* products is widely recommended due to their multidirectional beneficial action on health [1].

The most important finding of the present study, of high practical use, is the revelation that the consumption of AE under low-level and moderate chronic exposure to Cd offers effective protection from the destruction of the body status of Zn and Cu induced by this heavy metal. However, it is important to underline that apart from the protective (partial or complete) impact of AE against changes of various indices of the body status of Zn and Cu, the extract administration also influenced (decreased or increased) some indices of the body status of both bioelements unchanged by Cd alone. This mitigates the enthusiasm generated by our research results. Detailed analysis of the results showed that administration of the extract while feeding with the 1 mg Cd/kg diet completely prevented Zn and Cu deficiencies in internal organs in the young organism, and Cu deficiency in internal organs in the adult organism (after 17 months of the experiment), as well as the retention of both elements in the body due to lifetime Cd exposure. AE administration was also capable of completely counteracting the disorders in the body status of Zn caused by exposure to the 5 mg Cd/kg diet, but it decreased the concentration of this bioelement in the serum unaffected by Cd alone. The main negative effect of AE consumption was destroying the body status of Cu as a result of lifetime use of the extract in the case of moderate Cd treatment, consisting in its deficiency in internal organs, and the increase in the serum concentration of this bioelement. However, it is important to stress that the unfavourable changes of various indices of the body status of Zn and Cu due to AE consumption under exposure to Cd were transient and not marked.

The results of the ANOVA/MANOVA analysis allow us to recognise that the beneficial impact of AE administration under exposure to the 1 and 5 mg Cd/kg diets on the body status of Zn and Cu was caused by independent action of the extract ingredients and their interaction with Cd. The independent impact of AE might be related to the ability of ingredients present in chokeberry (cyanidin derivatives, fibers, and pectins) to chelate Zn and Cu [6,28,31,32], as well as to AE’s ability to inhibit MT biosynthesis in the liver, kidney, and duodenal tissue in the conditions of exposure to Cd. Although the administration of AE alone had no impact on the concentration of MT, its administration to the animals exposed to Cd markedly decreased this protein concentration and even increased the pool of non-MT-bound Cd in the liver, kidney, and duodenal tissue. The results of the ANOVA/MANOVA analysis clearly show that this was caused not only by the interaction of the extract ingredients with Cd, but also by their independent action. It is important to underline that our recent findings [11,58] indicate that the AE-caused decrease in the concentration of MT, responsible for Cd detoxification [22,37], as well as the increase in the pool of non-MT-bound Cd in the liver, is not associated with an increase in its toxicity. On the contrary, we have observed a protective effect of the extract on the liver of the female rats exposed to the 1 and 5 mg Cd/kg diets [11,58]. It has been revealed that polyphenolic compounds, including cyanidin derivatives (the main polyphenolic fraction of the AE) are able to chelate Zn and Cu [6,28,31,32]. Our more recent in vitro study has revealed that 0.1% AE, as well as cyanidin 3-galactoside present in the extract, are capable of chelating Zn and Cu (data in preparation for publication). The interactive impact of AE may be explained by its indirect action resulting from the influence on the body burden of Cd [7] due to the ability of the extract ingredients to bind Cd ions [6,28] and a decrease in MT concentration. Recently, we have reported that administration of the extract under exposure to 1 or 5 mg Cd/kg diets importantly decreased the body burden of this heavy metal, especially including its accumulation in the liver and kidneys, due to its decreased gastrointestinal absorption and increased urinary excretion [7]. Since the Cd content in the body was lower, the unfavourable effects of this toxic metal’s interactions with Zn and Cu were also less advanced. Thus, due to its ability to complex Zn and Cu ions and decrease Cd accumulation and MT biosynthesis, AE was able to counteract Cd-induced Zn and Cu retention in internal organs. Moreover, AE’s ability to protect against Cd-induced increase in the total pool of Cu in internal organs due to lifetime exposure to the 1 mg Cd/kg diet might result from the increased urinary excretion of this element noted in the Cd_1_ + AE group after 24 months of the experiment. The fact that the extract administration entirely prevented the deficiency in the total pool of Zn and Cu in internal organs of a young organism caused by low-level exposure to Cd shows that the content of Zn and Cu in the diet administered to young animals was sufficient to compensate the possible negative effect resulting from the ability of the extract ingredients to complex these bioelements in the gastrointestinal tract. It is important to emphasise that AE administration not only partially or entirely protected against changes in Zn and Cu concentrations and contents in tissues, but it also had a beneficial impact on the pool of MT-unbound bioelements in the liver, kidney, and duodenum. The Cd-induced decrease in the pool of MT-unbound Zn and Cu may indicate hampered gastrointestinal absorption of these bioelements and their insufficient availability to ensure the normal course of physiological processes dependent on them. Thus, revealing that the administration of AE maintains the pool of MT-unbound Zn and Cu at the proper level is another argument for the beneficial effect of the extract consumption under low-level and moderate exposure to Cd. The modifying impact of AE administration on bone tissue concentrations of Zn and Cu may be explained by the beneficial impact of the extract consumption on bone turnover that we have recently reported [5,9] and by the protection against Cd accumulation in the bone tissue (only at the moderate exposure) [7], and it might be related to the influence of the extract consumption on the body status of these bioelements. Wider discussion of the results of the present study on the impact of AE on the body status of Zn and Cu is impossible because of a lack of literature data on this subject.

We are not only aware of the achievements of the present study, but also its limitations. One of them is the inability, at this stage of our investigation, to exactly explain the mechanisms of the beneficial impact of AE on the body status of Zn and Cu. Moreover, while discussing the results of the present study on the possibility of the protective use of AE against disorders in the body status of Zn and Cu resulting from low-level and moderate exposure to Cd, we cannot ignore the fact that AE administration when feeding with 1 and 5 mg Cd/kg diets did not provide complete protection against all Cd-induced changes in the body status of Zn and Cu. In some cases, it also influenced the Cd alone-unchanged values of the estimated indices of the body status of these bioelements, resulting in an increase or decrease compared to the proper values of the control group and/or the values determined in the respective Cd group. At this stage of investigation, we are unable to explain the cause of these changes; however, they were innumerous and only occurred at some time points. Still, they limit the importance of our findings and thus require further study. The changes in Zn and Cu concentrations or contents in some tissues due to AE administration to female rats exposed to Cd may be related to the impact of the extract on the total body status of particular bioelements. It should also be taken into account when interpreting the results that the parameters describing the body status of Zn and Cu at particular time points were not evaluated in the same animals, but in various subgroups within particular experimental groups. This may also explain, at least to some extent, why some favourable effects of AE consumption under Cd exposure were or were not observed at some time points. We are also aware that since our findings come from an experimental female rat model, they only refer to the body status of Zn and Cu in females, and an analogous study in a male rat model is necessary. However, in spite of the limitations described above, the present study has provided reliable evidence that shows that the consumption of polyphenol-rich chokeberry products seems to be a promising strategy in the protection against Cd toxicity, and that efforts should be focused on investigation aimed at confirming their effectiveness in humans. First of all, it seems necessary to investigate the dependence between the consumption of these products and the health status of inhabitants of industrialised countries, including the indices of the body status of Zn and Cu.

## 5. Conclusions

In summary, the current study provides the first evidence that the consumption of AE under chronic low-level and moderate exposure to Cd may offer significant protection against most of the changes in the body status of Zn and Cu caused by this xenobiotic. However, it may also influence (increase or decrease) some of the indices of the metabolism of these bioelements unchanged by Cd. Although the unfavourable effects of the administration of the extract in the case of exposure to Cd were innumerous, transient, and not marked, and only occurred at some time points, they limit the importance of our findings and show that efforts should be focused on ruling out the risk of destroying the metabolism of Zn and Cu in the organism exposed to Cd due to the intake of *Aronia* products. Our results indicate that the influence of AE consumption on the body status of Zn and Cu under low-level and moderate exposure to Cd may be mediated by MT and that the beneficial effect of the extract may be related to its ability to maintain the pool of MT-unbound Zn and Cu at the proper level. Moreover, revealing that even long-term consumption of AE in the standard diet containing only trace amounts of Cd had no negative influence on the body status of Zn and Cu shows that incorporation of chokeberry products into the diet of healthy individuals with the aim of protecting their health should not disturb the body status of necessary bioelements such as Zn and Cu. On the basis of the findings of the present study, it seems that rational amounts of chokeberry products may be included in the daily diet unpolluted by Cd without the risk of destroying Zn and Cu metabolisms; however, their potential prophylactic use under exposure to Cd requires further study to exclude any unfavourable impact on the metabolism of these essential elements. These results, together with our previous findings on the beneficial impact of the administration of AE against some effects of the toxic action of Cd [5,7,9,10,11,58], allow us to conclude that *Aronia* berries and their products may be very promising natural agents for effective use in the prevention against various effects of this heavy metal action, including disorders in the metabolism of Zn and Cu. However, the occurrence of some unfavourable changes of various indices of the body status of Zn and Cu due to the consumption of AE under exposure to Cd somewhat reduces the enthusiasm arising from our studies, allowing us to conclude that products made from chokeberries may be promising preventive agents against various unfavourable actions of this xenobiotic. Thus, further studies on this subject are warranted to confirm their effectiveness and recognise health hazards related to their use.

## Figures and Tables

**Table 1:** Experimental model.

Group	Administration		Range of the Daily Intake During the 24-Month Administration	
	AE ^1^	Cd (1 or 5 mg Cd/kg) ^2^	AE (PF) (mg/kg b.w.) ^3^	Cd (μg/kg/b.w.) ^4^
Control	−	-		2.30–4.98
AE	+	-	67.4–146.6 (44.3–96.4)	2.25–4.95
Cd_1_	−	+(1 mg Cd/kg)		39.2–83.8
Cd_1_ + AE	+	+(1 mg Cd/kg)	67.2–154.7 (44.2–101.7)	37.5–84.9
Cd_5_	−	+(5 mg Cd/kg)		210.1–403.2
Cd_5_ + AE	+	+(5 mg Cd/kg)	63.1–150.3 (41.5–98.8)	200.2–401.9

^1^ AE (extract from the berries of *Aronia melanocarpa*) was administered as the only drinking fluid, in the form of 0.1% aqueous solution prepared by dissolving in redistilled water the powdered *Aronia* extract (Adamed Consumer Healthcare, Tuszyn, Poland; Certificate KJ 4/2010, Butch No. M100703), containing, according to the manufacturer, 65.74% of polyphenols (including 18.65% of anthocyanins). The powdered extract contained total polyphenols—612.40 ± 3.33 mg/g, total anthocyanins—202.28 ± 1.28 mg/g (cyanidin 3-*O*-β-galactoside—80.07 ± 1.05 mg/g, cyanidin 3-*O*-α-arabinoside—33.21 ± 0.01 mg/g, cyanidin 3-*O*-β-glucoside—3.68 ± 0.01 mg/g), total proanthocyanidins—129.87 ± 1.12 mg/g, total phenolic acids—110.92 ± 0.89 mg/g (chlorogenic acid—68.32 ± 0.08 mg/g), and total flavonoids—21.94 ± 0.98 mg/g [5]. The concentrations of zinc (Zn) and copper (Cu) in 0.1% AE reached 1.39 ± 0.04 μg/L and 0.803 ± 0.065 μg/L, respectively, whereas the cadmium (Cd) concentration was below the limit of detection (<0.05 μg/L [7]). ^2^ Cd diets were prepared by the addition, at their production stage, of appropriate amounts of cadmium chloride (CdCl_2_ × 2½ H_2_O) into the ingredients of the standard Labofeed H and B diets to achieve the concentrations of 1 and 5 mg Cd/kg. The concentration of Cd determined in our laboratory in the Labofeed H and B diets did not differ (reached by mean 1.09 ± 0.13 mg/kg and 4.92 ± 0.53 mg/kg) [7] and agreed with the values certified by the producer. ^3^ Data represent the range of the daily intake of AE and polyphenols (PF) throughout the 24-month study. Polyphenols intake was calculated assuming that the commercial extract contained 65.74% of these compounds (certified value). The intake of AE and polyphenols in the control group, Cd_1_ group, and Cd_5_ group was recognised to be 0. Detailed data on polyphenols intake in particular experimental groups have already been published [7]. ^4^ Data represent the range of the daily Cd intake throughout the 24-month study. Cd intake in the control group and AE group was calculated based on this metal concentration determined in the standard diet (0.0584 ± 0.0049 mg/kg) [7], while this metal intake in the groups exposed to Cd was calculated based on its concentration in the diet declared by the manufacturer (1 or 5 mg Cd/kg). Detailed data on Cd intake in particular groups have already been published [7]. “−” AE and/or Cd were not administered; “+” AE and/or Cd were administered.

**Table 2:** The intake of zinc (Zn) and copper (Cu) with diet in particular experimental groups ^1,2^.

Group	Experiment Duration			
	3 Months	10 Months	17 Months	24 Months
**Zn Intake (mg/kg b.w./24 h)**				
Control	16.596 ± 0.097	6.931 ± 0.201 **	6.345 ± 0.064 **	7.149 ± 0.069 **
AE	16.710 ± 0.265	7.037 ± 0.063 **	6.191 ± 0.117 **	7.138 ± 0.126 **
Cd_1_	16.282 ± 0.336	7.001 ± 0.015 **	6.361 ± 0.027 **	7.290 ± 0.143 **
Cd_1_ + AE	16.656 ± 0.039	7.060 ± 0.159 **	6.231 ± 0.036 **	7.408 ± 0.219 **
Cd_5_	16.175 ± 0.213	7.043 ± 0.076 **	6.176 ± 0.041 **	7.612 ± 0.259 **
Cd_5_ + AE	16.415 ± 0.144	7.191 ± 0.054 **	6.227 ± 0.019 **	7.557 ± 0.241 **
**Cu Intake (mg/kg b.w./24 h)**				
Control	2.678 ± 0.038	1.155 ± 0.033 **	1.026 ± 0.006 **	1.194 ± 0.004 **
AE	2.626 ± 0.042	1.186 ± 0.012 **	1.032 ± 0.019 **	1.190 ± 0.021 **
Cd_1_	2.638 ± 0.021	1.159 ± 0.003 **	1.032 ± 0.001 **	1.215 ± 0.024 **
Cd_1_ + AE	2.702 ± 0.032	1.177 ± 0.026 **	1.004 ± 0.019 **	1.235 ± 0.036 **
Cd_5_	2.616 ± 0.011	1.174 ± 0.013 **	1.026 ± 0.006 **	1.269 ± 0.043 **
Cd_5_ + AE	2.614 ± 0.008	1.198 ± 0.009 **	1.019 ± 0.008 **	1.260 ± 0.040 **

^1^ The intake of Zn and Cu was calculated based on these bioelements’ concentrations in the Labofeed diets declared by the manufacturer. The Labofeed H diet (administered throughout the first 3 months of the study) contained 210 mg Zn/kg and 33 mg Cu/kg, whereas the Labofeed B diet (used thereafter), contained 150 mg Zn/kg and 25 mg Cu/kg. ^2^ Data represent mean ± SE intake of Zn and Cu for 32, 24, 16, and eight rats during 3, 10, 17, and 24 months of the experiment, respectively. ** *p* < 0.01 (ANOVA, Duncan’s multiple range test) compared to the intake during the first 3 months.

**Table 3:** Effect of the extract from the berries of *Aronia melanocarpa* (AE) and/or cadmium (Cd) on the degree of zinc (Zn), copper (Cu), and Cd binding to metallothionein (MT) in the liver ^1,2,3^.

Metals Binding to MT in the Liver	Effect of AE Alone	1 mg Cd/kg Diet + AE			5 mg Cd/kg Diet + AE		
		Effect of Cd Alone	Cd + AE		Effect of Cd Alone	Cd + AE	
			Effect of Cd + AE	Effect of AE		Effect of Cd + AE	Effect of AE
	**3 months**						
Zn/(MT × 7)	↔	↓ 48%	↔	↗ 2.1-fold	↓ 53%	↔	↗ 2.2-fold
Cu/(MT × 12)	↔	↓ 47%	↔	↗ 2.1-fold	↓ 57%	↔	↗ 2.2-fold
Cd/(MT × 7)	↔	↔	↔	↔	↑ 11.6-fold	↑ 21.3-fold	↗ 84%
Me/(Me-MT)	↔	↓ 48%	↔	↗ 2.1-fold	↓ 53%	↔	↗ 2.2-fold
	**10 months**						
Zn/(MT × 7)	↔	↓ 55%	↔	↗ 2.7-fold	↓ 58%	↔	↗ 81%
Cu/(MT × 12)	↔	↓ 55%	↔	↗ 2.8-fold	↓ 61%	↓ 29%	↗ 85%
Cd/(MT × 7)	↔	↔	↑ 9.4-fold	↗ 2.4-fold	↑ 25.8-fold	↑ 42.4-fold	↗ 64%
Me/(Me-MT)	↔	↓ 55%	↔	↗ 2.7-fold	↓ 57%	↔	↗ 81%
	**17 months**						
Zn/(MT × 7)	↓ 30%	↓ 57%	↓ 36%	↔	↓ 63%	↔	↗ 2.6-fold
Cu/(MT × 12)	↓ 27%	↓ 60%	↓ 18%	↗ 2.0-fold	↓ 58%	↔	↗ 2.5-fold
Cd/(MT × 7)	↔	↔	↔	↔	↑ 68.7-fold	↑ 136-fold	↗ 98%
Me/(Me-MT)	↓ 30%	↓ 57%	↓ 35%	↗ 52%	↓ 61%	↔	↗ 2.6-fold
	**24 months**						
Zn/(MT × 7)	↓ 34%	↓ 52%	↓ 24%	↗ 57%	↓ 69%	↔	↗ 3.1-fold
Cu/(MT × 12)	↓ 32%	↓ 54%	↓ 26%	↗ 59%	↓ 71%	↔	↗ 3.1-fold
Cd/(MT × 7)	↔	↔	↔	↔	↑ 66-fold	↑ 165-fold	↗ 2.5-fold
Me/(Me-MT)	↓ 34%	↓ 52%	↓ 24%	↗ 57%	↓ 68%	↔	↗ 3.1-fold

^1^ The rats received the 0.1% aqueous AE or not and Cd in their diet at the concentration of 0, 1, and 5 mg/kg. ^2^ In this table, only statistically significant (*p* < 0.05) changes compared to the control group (↓, percentage decrease; ↑, percentage increase or a factor of increase) and the respective group that received Cd alone (↗, percentage increase or a factor of increase) are indicated. ↔, without change (*p* > 0.05) compared to the control group; ↔, without change (*p* > 0.05) compared to the respective group treated with Cd alone. The Zn/(MT × 7) in the liver of the control group reached 2.182 ± 0.222, 2.927 ± 0.166, 2.749 ± 0.259, and 3.334 ± 0.403, after 3, 10, 17, and 24 months, respectively, whereas the Cu/(MT × 12) was 0.249 ± 0.026, 0.238 ± 0.017, 0.218 ± 0.023, and 0.324 ± 0.036, respectively. The Cd/(MT × 7) reached 0.0017 ± 0.0002, 0.0013 ± 0.00005, 0.0006 ± 0.00006, and 0.0007 ± 0.0001, while the Me/(Me-MT) was 1.297 ± 0.062, 2.888 ± 0.214, 3.044 ± 0.442, and 2.468 ± 0.243 after 3, 10, 17, and 24 months, respectively. ^3^ An increase in the Zn/(MT × 7), Cu/(MT × 12), Cd/(MT × 7), or Me/Me-MT ratios compared to the control group or any other experimental group indicates a rise in the pool of MT-unbound metals (Zn, Cu, Cd, or all of the metals, respectively), while a decrease in these ratios reflects a drop in the pool of non-MT-bound metals. Zn/(MT × 7), the pool of MT-unbound Zn; Cu/(MT × 12), the pool of MT-unbound Cu; Cd/(MT × 7), the pool of MT-unbound Cd; Me/(Me-MT), the pool of MT-unbound metals (Zn, Cu, and Cd).

**Table 4:** Effect of the extract from the berries of *Aronia melanocarpa* (AE) and/or cadmium (Cd) on the degree of zinc (Zn), copper (Cu), and Cd binding to metallothionein (MT) in the kidney ^1,2,3^.

Metals Binding to MT in the Kidney	Effect of AE Alone	1 mg Cd/kg Diet + AE			5 mg Cd/kg Diet + AE		
		Effect of Cd Alone	Cd + AE		Effect of Cd Alone	Cd + AE	
			Effect of Cd + AE	Effect of AE		Effect of Cd + AE	Effect of AE
	**3 months**						
Zn/(MT × 7)	↔	↔	↔	↗ 57%	↔	↓ 33%	↗ 73%
Cu/(MT × 12)	↔	↓ 40%	↔	↗ 2.1-fold	↔	↔	↔
Cd/(MT × 7)	↔	↑ 7.4-fold	↑ 8.5-fold	↔	↑ 25.2-fold	↑ 39.1-fold	↗ 55%
Me/(Me-MT)	↔	↔	↔	↗ 65%	↔	↔	↗ 56%
	**10 months**						
Zn/(MT × 7)	↓ 22%	↓ 41%	↓ 24%	↗ 29%	↓ 47%	↓ 35%	↔
Cu/(MT × 12)	↓ 20%	↓ 53%	↓ 29%	↗ 50%	↓ 28%	↓ 45%	↔
Cd/(MT × 7)	↔	↑ 13.3-fold	↑ 33.6-fold	↔	↑ 47.9-fold	↑ 51.3-fold	↔
Me/(Me-MT)	↓ 20%	↓ 42%	↓ 23%	↗ 32%	↓ 39%	↓ 31%	↔
	**17 months**						
Zn/(MT × 7)	↔	↓ 26%	↓ 24%	↗ 67%	↑ 48%	↑ 29%	↘ 13%
Cu/(MT × 12)	↔	↓ 53%	↔	↗ 2.2-fold	↓ 46%	↔	↗ 91%
Cd/(MT × 7)	↔	↔	↑ 33.6-fold	↔	↑ 139-fold	↑ 223-fold	↗ 61%
Me/(Me-MT)	↔	↓ 29%	↑ 23%	↗ 26%	↓ 21%	↑ 42%	↗ 81%
	**24 months**						
Zn/(MT × 7)	↔	↓ 42%	↓ 29%	↔	↓ 56%	↓ 30%	↗ 90%
Cu/(MT × 12)	↔	↓ 34%	↔	↔	↓ 58%	↓ 32%	↗ 62%
Cd/(MT × 7)	↔	↑ 12.1-fold	↑ 17.1-fold	↔	↑ 41.1-fold	↑ 51.3-fold	↗ 25%
Me/(Me-MT)	↔	↓ 39%	↓ 26%	↔	↓ 50%	↓ 24%	↗ 54%

^1^ The rats received the 0.1% aqueous AE or not and Cd in their diet at the concentration of 0, 1, and 5 mg/kg. ^2^ In this table, only statistically significant (*p* < 0.05) changes compared to the control group (↓, percentage decrease; ↑, percentage increase or a factor of increase) and the respective group that received Cd alone (↘, percentage decrease; ↗, percentage increase or a factor of increase) are indicated. ↔, without change (*p* > 0.05) compared to the control group; ↔, without change (*p* > 0.05) compared to the respective group treated with Cd alone. The Zn/(MT × 7) in the kidney of the control group reached 1.039 ± 0.05, 1.364 ± 0.08, 1.286 ± 0.069, and 1.830 ± 0.106, after 3, 10, 17, and 24 months, respectively, whereas the Cu/(MT × 12) was 0.257 ± 0.016, 0.232 ± 0.016, 0.284 ± 0.015, and 0.289 ± 0.022. The Cd/(MT × 7) reached 0.001 ± 0.0001, 0.002 ± 0.0002, 0.001 ± 0.0001, and 0.003 ± 0.0003, while the Me/(Me-MT) was 1.297 ± 0.062, 1.598 ± 0.095, 1.571 ± 0.083, and 2.122 ± 0.125 after 3, 10, 17, and 24 months, respectively. ^3^ An increase in the Zn/(MT × 7), Cu/(MT × 12), Cd/(MT × 7), or Me/Me-MT ratios compared to the control group or any other experimental group indicates a rise in the pool of MT-unbound metals (Zn, Cu, Cd, or all of the metals, respectively), while a decrease in these ratios reflects a drop in the pool of non-MT-bound metals. Zn/(MT × 7), the pool of MT-unbound Zn; Cu/(MT × 12), the pool of the MT-unbound Cu; Cd/(MT × 7), the pool of the MT-unbound Cd; Me/(Me-MT), the pool of MT-unbound metals (Zn, Cu, and Cd).

**Table 5:** Effect of the extract from the berries of *Aronia melanocarpa* (AE) and/or cadmium (Cd) on the degree of zinc (Zn), copper (Cu), and Cd binding to metallothionein (MT) in the duodenum ^1,2,3^.

Metals Binding to MT in the Duodenum	Effect of AE Alone	1 mg Cd/kg Diet + AE			5 mg Cd/kg Diet + AE			
		Effect of Cd Alone	Cd + AE		Effect of Cd Alone	Cd + AE	
			Effect of Cd + AE	Effect of AE		Effect of Cd + AE	Effect of AE
	**3 months**						
Zn/(MT × 7)	↔	↑ 64%	↔	↗ 81%	↑ 81%	↔	↗ 62%
Cu/(MT × 12)	↔	↓ 39%	↔	↗ 66%	↓ 41%	↔	↗ 42%
Cd/(MT × 7)	↔	↑ 2.1-fold	↑ 3.8-fold	↗ 78%	↑ 7.2-fold	↑ 10.7-fold	↗ 50%
Me/(Me-MT)	↔	↔	↔	↗ 65%	↔	↔	↗ 56%
	**10 months**						
Zn/(MT × 7)	↔	↓ 43%	↓ 28%	↔	↓ 55%	↓ 26%	↗ 64%
Cu/(MT × 12)	↔	↓ 47%	↓ 32%	↔	↓ 58%	↓ 34%	↗ 57%
Cd/(MT × 7)	↔	↑ 6.2-fold	↑ 9.7-fold	↗ 57%	↑ 11.6-fold	↑ 19.9-fold	↗ 72%
Me/(Me-MT)	↓ 22%	↓ 42%	↓ 23%	↗ 32%	↓ 39%	↓ 31%	↔
	**17 months**						
Zn/(MT × 7)	↓ 28%	↓ 55%	↔	↗ 2.4-fold	↓ 62%	↓ 29%	↗ 88%
Cu/(MT × 12)	↓ 23%	↓ 48%	↔	↗ 2.1-fold	↓ 58%	↓ 24%	↗ 80%
Cd/(MT × 7)	↔	↑ 5.3-fold	↑ 12.5-fold	↗ 2.4-fold	↑16.5-fold	↑ 22.6-fold	↗ 37%
Me/(Me-MT)	↔	↓ 29%	↑ 23%	↗ 74%	↓ 21%	↓ 42%	↗ 81%
	**24 months**						
Zn/(MT × 7)	↓ 22%	↓ 52%	↓ 49%	↔	↓ 58%	↓ 20%	↗ 90%
Cu/(MT × 12)	↓ 20%	↓ 48%	↓ 46%	↔	↓ 56%	↔	↗ 95%
Cd/(MT × 7)	↔	↔	↑ 3.9-fold	↔	↑7.5-fold	↑ 15.4-fold	↗ 2-fold
Me/(Me-MT)	↔	↓ 39%	↓ 26%	↔	↓ 50%	↓ 24%	↗ 54%

^1^ The rats received the 0.1% aqueous AE or not and Cd in their diet at the concentration of 0, 1, and 5 mg/kg. ^2^ In this table, only statistically significant (*p* < 0.05) changes compared to the control group (↓, percentage decrease; ↑, percentage increase or a factor of increase) and the respective group that received Cd alone (↗, percentage increase or a factor of increase) are indicated. ↔, without change (*p* > 0.05) compared to the control group; ↔, without change (*p* > 0.05) compared to the respective group treated with Cd alone. The Zn/(MT × 7) in the duodenum of the control group reached 3.490 ± 0.160, 2.792 ± 0.207, 2.915 ± 0.424, and 2.375 ± 0.234, after 3, 10, 17, and 24 months, respectively, whereas the Cu/(MT x 12) was reached 0.140 ± 0.005, 0.095 ± 0.007, 0.128 ± 0.017, and 0.091 ± 0.009. The Cd/(MT × 7) reached 0.003 ± 0.0002, 0.0012 ± 0.0001, 0.001 ± 0.0001, and 0.0017 ± 0.0002, while the Me/(Me-MT) was 3.633 ± 0.164, 2.888 ± 0.214, 3.044 ± 0.442, and 2.468 ± 0.243 after 3, 10, 17, and 24 months, respectively. ^3^ An increase in the Zn/(MT × 7), Cu/(MT × 12), Cd/(MT × 7), or Me/Me-MT ratios compared to the control group or any other experimental group indicates a rise in the pool of MT-unbound metals (Zn, Cu, Cd, or all of the metals, respectively), while a decrease in these ratios reflects a drop in the pool of non-MT-bound metals. Zn/(MT × 7), the pool of MT-unbound Zn; Cu/(MT × 12), the pool of MT-unbound Cu; Cd/(MT × 7), the pool of MT-unbound Cd; Me/(Me-MT), the pool of MT-unbound metals (Zn, Cu, and Cd).

## Supplementary Materials

The following are available online at www.mdpi.com/2072-6643/9/12/1374/s1, Supplementary Material—Zinc, Supplementary Material—Copper; Table S1: Analytical quality of zinc (Zn) and copper (Cu) measurements in certified reference materials, Table S2: Effect of the extract from the berries of *Aronia melanocarpa* (AE) on cadmium (Cd) concentration in the liver and kidney of rats exposed to this toxic metal, Table S3: The daily intake of zinc (Zn) and copper (Cu) with diet in particular experimental groups during the 5-day balance study, Table S4: Main and interactive effects of cadmium (Cd) and the extract from the berries of *Aronia melanocarpa* (AE) on apparent absorption (Abs_Zn_), retention in the body (Ret_Zn_), and faecal (FE_Zn_) and urinary (UE_Zn_) excretion of zinc (Zn), Table S5: Main and interactive effects of cadmium (Cd) and the extract from the berries of *Aronia melanocarpa* (AE) on zinc (Zn) concentration in the serum and tissues of rats exposed to the 1 mg Cd/kg diet, Table S6: Main and interactive effects of cadmium (Cd) and the extract from the berries of *Aronia melanocarpa* (AE) on zinc (Zn) concentration in the serum and tissues of rats exposed to the 5 mg Cd/kg diet, Table S7: Main and interactive effects of cadmium (Cd) and the extract from the berries of *Aronia melanocarpa* (AE) on zinc (Zn) content in internal organs, Table S8: Main and interactive effects of cadmium (Cd) and the extract from the berries of *Aronia melanocarpa* (AE) on apparent absorption (Abs_Cu_), retention in the body (Ret_Cu_), and faecal (FE_Cu_) and urinary (UE_Cu_) excretion of copper (Cu), Table S9: Main and interactive effects of cadmium (Cd) and the extract from the berries of *Aronia melanocarpa* (AE) on copper (Cu) concentration in the serum and tissues of rats exposed to the 1 mg Cd/kg diet, Table S10: Main and interactive effects of cadmium (Cd) and the extract from the berries of *Aronia melanocarpa* (AE) on copper (Cu) concentration in the serum and tissues of rats exposed to the 5 mg Cd/kg diet, Table S11: Main and interactive effects of cadmium (Cd) and the extract from the berries of *Aronia melanocarpa* (AE) on copper (Cu) content in internal organs, Table S12: Main and interactive effects of cadmium (Cd) and the extract from the berries of *Aronia melanocarpa* (AE) on metallothionein (MT) concentration in the liver and the degree of zinc (Zn), copper (Cu), and Cd binding to this protein, Table S13: Main and interactive effects of cadmium (Cd) and the extract from the berries of *Aronia melanocarpa* (AE) on metallothionein (MT) concentration in the kidney and the degree of zinc (Zn), copper (Cu), and Cd binding to this protein, Table S14: Main and interactive effects of cadmium (Cd) and the extract from the berries of *Aronia melanocarpa* (AE) on metallothionein (MT) concentration in the duodenum and the degree of zinc (Zn), copper (Cu), and Cd binding to this protein, Figure S1: The body retention of zinc (Ret_Zn_) and copper (Ret_Cu_) in particular experimental groups, Figure S2: Zinc (Zn) content in the kidney, liver, and heart in particular experimental groups, Figure S3: Zinc (Zn) content in the spleen and brain in particular experimental groups, Figure S4: Copper (Cu) content in the kidney, liver, and heart in particular experimental groups, Figure S5: Copper (Cu) content in the spleen and brain in particular experimental groups.

## Abbreviations

AAS	atomic absorption spectrometry
Abs_Cu_	apparent absorption of copper
Abs_Zn_	apparent absorption of zinc
AE	extract from the berries of *Aronia melanocarpa*
*A. melanocarpa*	*Aronia melanocarpa*
Cd	cadmium
CdCl_2_ × 2½ H_2_O	cadmium chloride
Cd/(MT × 7)	the pool of metallothionein-unbound cadmium
Cu	copper
Cu/(MT × 12)	the pool of metallothionein-unbound copper
CV	coefficient of variation
F AAS	flame atomic absorption spectrometry
GF AAS	flameless atomic absorption spectrometry with electrothermal atomisation in a graphite furnace
FE_Cu_	faecal copper excretion
FE_Zn_	faecal zinc excretion
HCl	hydrochloric acid
HNO_3_	nitric acid
Me	metal (zinc, copper, or cadmium)
Me/Me-MT	the total pool of metallothionein-unbound metals
MT	metallothionein
NaCl	sodium chloride
Ret_Cu_	copper retention in the body
Ret_Zn_	zinc retention in the body
SD	standard deviation
SE	standard error
UE_Cu_	urinary copper excretion
UE_Zn_	urinary zinc excretion
Zn	zinc
Zn/(MT × 7)	the pool of metallothionein-unbound zinc
