# Supplementary material for: Effect of an Extract from Aronia melanocarpa L. Berries on the Body Status of Zinc and Copper under Chronic Exposure to Cadmium: An In Vivo Experimental Study
Source: Nutrients. 2017 Dec 19;9(12):1374. doi: 10.3390/nu9121374 (PMC5748824; doi:10.3390/nu9121374)
Supplement: Supplementary file 1 [file nutrients-09-01374-s001.pdf]

nutrients

Supplementary Materials

Effect of an Extract from *Aronia melanocarpa* L. Berries on the Body Status of Zinc and Copper under Chronic Exposure to Cadmium: An in Vivo Experimental Study

Sylwia Borowska *, Małgorzata M. Brzóska *, Małgorzata Gałażyn-Sidorczuk, Joanna Rogalska

Department of Toxicology, Medical University of Bialystok, Adama Mickiewicza 2C street, 15-222 Bialystok, Poland; malgorzata.galazyn-sidorczuk@umb.edu.pl (M.G.-S.); joanna.rogalska@umb.edu.pl (J.R.)

* Correspondence: sylwia.borowska@umb.edu.pl (S.B.), malgorzata.brzoska@umb.edu.pl (M.M.B.); Tel.: +48-85-748-56-04 (S.B. & M.M.B.); Fax: +48-85-748-58-34 (S.B. & M.M.B.)

Supplementary Material–Zinc

Zinc 1

The administration of a polyphenol-rich aronia berries extract (AE) alone resulted in a decrease in the apparent absorption (Abs_{Zn}) and retention (Ret_{Zn}) of zinc (Zn), and an increase in this bioelement faecal excretion (FE_{Zn}) after 10 months, as well as in an increase in its urinary excretion (UE_{Zn}) after 17 months of the experiment (Figures 1 and S1).

The administration of AE during 3-month exposure to the 1 mg Cd/kg diet increased the Abs_{Zn} and Ret_{Zn} and decreased the FE_{Zn}, unaffected by cadmium (Cd) alone, making them different compared to the respective group treated with Cd alone and the control group (Figures 1 and S1). The administration of AE under the 10-month exposure to the 1 mg Cd/kg diet had no influence on the Cd-induced changes in the Abs_{Zn}, Ret_{Zn}, and FE_{Zn} (Figures 1 and S1). Moreover, the consumption of AE during the 24 month-exposure to the 1 mg Cd/kg diet increased the UE_{Zn}, unaffected by Cd alone, compared to the Cd_1 group and control group (Figure 1). The administration of AE under the 3-month exposure to the 5 mg Cd/kg diet had no impact on the Cd-induced changes in the Abs_{Zn}, Ret_{Zn}, and FE_{Zn}, as well as on the Cd-decreased UE_{Zn} after 10 months. Moreover, the AE consumption under the 24-month exposure to the 5 mg Cd/kg diet decreased the UE_{Zn} compared to the Cd_5 group (Figure 1).

Zinc 2

The administration of AE alone for up to 24 months resulted in an increase in the heart and decrease in the stomach Zn concentration after 3 months, a drop in the duodenal tissue after 17 months, and an increase in the brain and decrease in the femoral muscle concentration of this element after 24 months (Figures 2–6).

The consumption of AE under exposure to the 1 mg Cd/kg diet resulted in a decrease (kidney after 3 months, heart after 10 months, heart and liver after 17 months, and stomach and duodenum after 24 months; Figures 2, 3, and 5) or increase (brain after 3 months, spleen after 17 months, and bone tissue at the femoral distal epiphysis after 24 months; Figures 2–4) in Cd alone-unchanged Zn concentration in some tissues compared to the control group (in the case of liver, brain, and duodenum also compared to the Cd_1 group).

AE administration under the treatment with the diet containing 5 mg Cd/kg modified (decreased or increased) Cd alone-unchanged Zn concentration in the serum and various tissues in comparison to the control group and/or Cd_5 group at different time points (Figures 2–4). The extract consumption

led to a decrease (kidney and bone tissue at the femoral diaphysis after 3 months, spleen after 10 months, brain after 24 months; Figures 2–4) or increase (heart after 3 months, kidney after 10 months, femoral muscle after 17 months, kidney and liver after 24 months; Figures 2–4) in Cd alone-unchanged Zn concentration in some tissues compared to the control group (in the case of bone tissue at the femoral diaphysis and kidney concentration after 10 months also compared to the Cd_5 group). AE co-administration had no impact on the exposure to the 5 mg Cd/kg diet-induced decrease in the heart after 10 and 17 months (Figure 3), duodenum and bone tissue at the femoral distal epiphysis after 17 months (Figures 4 and 5), and stomach after 24 months (Figure 3), as well as the 17-month exposure to this xenobiotic-caused increase in the concentration of Zn in the spleen and kidney (Figure 2).

Zinc 3

The administration of AE under exposure to the 1 and/or 5 mg Cd/kg diet changed (increased or decreased), Cd-unaffected content of Zn in some internal organs (liver, heart, spleen, and brain) in comparison to the control/or relevant Cd group (Figures 6, S2, and S3). The administration of the AE under exposure to the 1 mg Cd/kg diet increased the unchanged by this toxic metal Zn content in brain after 3 months, compared to the control (by 21%) and the group treated with Cd alone (by 16%) and in spleen after 17 months, compared to the Cd_1 group (by 30%). Moreover, the administration of the extract to the animals fed for 3 months with the diet containing 5 mg Cd/kg resulted in an increase, compared to the control group, in the Cd-unchanged content of Zn in heart, spleen, and brain (by 13%, 17%, and 14%, respectively; Figures S2 and S3). Brain content of Zn in rats receiving the AE under the 24-month low-level and moderate treatment with Cd, as well as liver Zn content and the sum of its content in the liver and kidneys in the case of the 5 mg Cd/kg diet were lower (by 13–14%) compared to the respective Cd group; however, this bioelement content in these organs was within the range of the control group (Figures S2 and S3).

Supplementary Material–Copper

Copper 1

The administration of AE alone decreased the apparent absorption (Abs_{Cu}) and retention (Ret_{Cu}) of copper (Cu) and increased its faecal excretion (FE_{Cu}) after 10 months (Figures 7 and S1).

The administration of AE under 10-month exposure to the 1 mg Cd/kg diet declined the Abs_{Cu} and Ret_{Cu}, unaffected by Cd alone, compared to the group treated with Cd alone and the control group, and it increased the FE_{Cu} (Figures 7 and S1). Moreover, the co-administration of the 1 mg Cd/kg diet and AE increased the UE_{Cu} compared to the control group after 17 months and compared to the control group and Cd_1 group after 24 months (Figure 7).

Copper 2

The administration of AE alone resulted in an increase in the heart and decrease in the serum, spleen and stomach concentration of Cu after 3 months, a decline in the duodenal tissue after 10 months, a decrease in the duodenal tissue and kidney, as well as an increase in the bone tissue at the femoral distal epiphysis after 17 months (Figures 5 and 8–11).

Compared to the control group, the administration of AE under exposure to the 1 mg Cd/kg diet declined this heavy metal-unaffected Cu concentration in the serum after 3 months and did not influence its increased serum concentration after 17 months (Figure 11). The consumption of AE under the low exposure to Cd resulted in a decrease or increase in Cu concentration in some tissues (liver, spleen, brain, stomach, duodenum, femoral muscle, bone tissue) unchanged by Cd alone compared to the control group and/or the Cd_1 group at some time points (Figures 8–10). The extract intake caused a decrease (bone tissue at the femoral distal epiphysis after 3 months, duodenum after 17 months; Figures 5 and 10) in Cd alone-unchanged Cu concentration in some tissues compared to the control group (in the case of duodenum after 17 months also compared to the Cd_1 group), as well as in the case of femoral muscle (after 3 months) and brain (after 24 months) compared only to Cd_1

group (Figures 9 and 10). Moreover, the co-administration of AE resulted in an increase in Cu concentration in the brain and stomach after 10 months (Figures 8 and 9), bone tissue at the femoral distal epiphysis after 17 months (Figure 10) and spleen after 24 months (Figure 8) compared to the control group (in the case of brain after 10 months, stomach and bone tissue at the femoral distal epiphysis also compared to Cd_1 group), as well as in the case of brain after 3 months and liver after 17 months compared only to Cd_1 group (Figures 8 and 9). The extract administration had no impact on the decreased stomach Cu concentration after 3 and 17 months of exposure to the 1 mg Cd/kg diet (Figure 9).

The administration of AE to the animals fed with the 5 mg Cd/kg diet influenced the serum Cu concentration at all time-points (Figure 11). Three- and 10-month intake of the extract decreased the Cd alone-unchanged Cu concentration in the serum compared to the control group and compared to the Cd_5 group after the shorter co-administration. Seventeen-month consumption of the extract partially protected from this xenobiotic-caused increase in the serum concentration of this bioelement, while its 24-month intake increased the serum Cu level compared to the control group and Cd_5 group (Figure 11). The extract consumption under the moderate exposure to Cd increased, compared to the control group and Cd_5 group, the Cd alone-unaffected Cu concentration in the heart after 3 months (Figure 9) and duodenum after 24 months (Figure 5). Moreover, the 24-month administration of the AE to the animals exposed to the 5 mg Cd/kg diet increased, but only compared to the Cd_5 group, the Cd-unchanged Cu concentration in the spleen (Figure 8) and heart (Figure 9). The extract administration did not provide protection regarding the exposure to the 5 mg Cd/kg diet-induced decrease in Cu concentration in the spleen after 3 months (Figure 8), stomach after 3 and 17 months (Figure 9), duodenum after 17 months (Figure 5), and bone tissue at the femoral distal epiphysis after 3 months (Figure 10), as well as the 3-month treatment-caused increase in this bioelement concentration in the bone tissue at the femoral diaphysis (Figure 10).

Copper 3

The extract consumption decreased the Cd-unchanged sum of the content of this element in the liver and kidneys and its total pool after 3 and 24 months, compared to the control group and the Cd_5 group, except for the total Cu pool in internal organs after 3 months, which was lower only compared to the Cd_5 group (Figures 11 and S4). Moreover, the administration of AE under exposure to the 1 and/or 5 mg Cd/kg diet at some time points changed (increased or decreased) the content of Cu in some internal organs (liver, spleen and brain), unaffected by this toxic metal, in comparison to the control and/or appropriate Cd group (Figures 11, S4, and S5).

Table S1. Analytical quality of zinc (Zn) and copper (Cu) measurements in certified reference materials.

Kind of Certified Reference Material	Element	Reference Values	Noticed Values [1]	Recovery	Precision (CV) [2]
Trace Elements Serum L-1 LOT (no. 0903106; Sero, Billingstad, Norway)	Zn	1667 – 1809 μg/L (mean 1738 μg/L)	1697 ± 23.9 μg/L	98%	1.4%
	Cu	1607 – 1775 μg/L (mean 1691 μg/L)	1672 ± 158 μg/L	99%	9.4%
Trace Elements Urine L-2 LOT (no. 1011645; Sero, Billingstad, Norway)	Zn	1338 ± 269 μg/L	1267 ± 27.3 μg/L	95%	2.2%
	Cu	22 μg/L	23.1 ± 1.7 μg/L	105%	7.4%
Standard Reference Material Bovine Liver (no. 1577b; National Institute of Standards and Technology, Gaithersburg, MD, USA)	Zn	127 ± 16 μg/g	123.7 ± 14.3 μg/g	97%	1.2%
	Cu	184 ± 15 μg/g	177 ± 13 μg/g	96%	7.3%
Certified Reference Material BCR Pig Kidney (BCR-186; Institute for Reference Materials and Measurements, Geel, Belgium)	Zn	128 ± 3 μg/g	123.5 ± 4.1 μg/g	96%	3.3%
	Cu	31.9 ± 0.4 μg/g	30.7 ± 0.31 μg/g	96%	1.0%
Standard Reference Bone Ash (no. 1400; National Institute of Standards and Technology, Gaithersburg, MD, USA)	Zn	181 ± 3 μg/g	187.2 ± 3.8 μg/g	103%	2.0%
	Cu	2.3 μg/g	2.41 ± 0.19 μg/g	105%	7.9%

[1] Data are represented as mean ± SE for three measurements. [2] Precision of measurements is expressed as a coefficient of variation (CV).

Table S2. Effect of the extract from the berries of *Aronia melanocarpa* (AE) on cadmium (Cd) concentration in the liver and kidney of rats exposed to this toxic metal. [1,2]

Organ	Effect of Cd Alone	Cd_1 + AE		Effect of Cd Alone	Cd_5 + AE	
		Effect of Cd + AE	Effect of AE		Effect of Cd + AE	Effect of AE
**3 months**						
**Liver**	↑ 4.1-fold***	↑ 2.8-fold***	↘ 33%†††	↑ 26-fold***	↑ 21-fold***	↘ 19%†††
**Kidney**	↑ 9.4-fold***	↑ 6.7-fold***	↘ 29%†††	↑ 37-fold***	↑ 32-fold***	↘ 13%†††
**10 months**						
**Liver**	↑ 8.7-fold***	↑ 7.8-fold***	↔	↑ 70-fold***	↑ 63-fold***	↘ 11%†
**Kidney**	↑ 22-fold***	↑ 20-fold***	↔	↑ 96-fold***	↑ 89-fold***	↘ 7%†
**17 months**						
**Liver**	↑ 15-fold***	↑ 14-fold***	↔	↑ 175-fold***	↑ 132-fold***	↘ 25%††
**Kidney**	↑ 26-fold***	↑ 28-fold***	↔	↑ 229-fold***	↑ 206-fold***	↘ 10%††
**24 months**						
**Liver**	↑ 26-fold***	↑ 16-fold***	↘ 38%††	↑ 197-fold***	↑ 178-fold***	↘ 10%†
**Kidney**	↑ 24-fold***	↑ 24-fold***	↔	↑ 95-fold***	↑ 85-fold***	↘ 11%†

[1] The rats received 0.1% aqueous AE or not and Cd in diet at the concentration of 0, 1, and 5 mg/kg. [2] Detailed data on Cd concentration in the liver and kidney in all experimental groups have already been presented [7]. In this table only changes compared to the control group (↑, a factor of increase, $^{***}p < 0.001$), and the respective group that received Cd alone ($^{†}p < 0.05$, $^{††}p < 0.01$, $^{†††}p < 0.001$; ↘, a factor of decrease) are indicated. ↔, without change ($p > 0.05$) compared to the respective group treated with Cd alone. Cd concentration in the liver in the control group reached 0.035 ± 0.003 μg/g, 0.023 ± 0.001 μg/g, 0.014 ± 0.002 μg/g, and 0.014 ± 0.001 μg/g after 3, 10, 17, and 24 months, respectively, whereas its kidney concentration was 0.037 ± 0.003 μg/g, 0.050 ± 0.002 μg/g, 0.047 ± 0.003 μg/g, and 0.084 ± 0.013 μg/g, respectively.

Table S3. The daily intake of zinc (Zn) and copper (Cu) with diet in particular experimental groups during the 5-day balance study. [1,2,3]

Group	Experiment duration			
	3 months	10 months	17 months	24 months
**Zn intake (mg/24 h)**				
**Control**	**5.182 ± 0.073**	**2.994 ± 0.087****	**3.356 ± 0.061****	**4.196 ± 0.071****
**AE**	**5.163 ± 0.082**	**3.060 ± 0.070****	**3.276 ± 0.061****	**4.054 ± 0.071****
**Cd_1**	**5.088 ± 0.099**	**3.079 ± 0.051****	**3.366 ± 0.050****	**4.279 ± 0.084****
**Cd_1 + AE**	**5.003 ± 0.059**	**3.131 ± 0.070****	**3.297 ± 0.059****	**4.252 ± 0.126****
**Cd_5**	**5.095 ± 0.067**	**3.120 ± 0.034****	**3.268 ± 0.046****	**4.354 ± 0.148****
**Cd_5 + AE**	**5.187 ± 0.045**	**3.009 ± 0.023****	**3.295 ± 0.024****	**4.187 ± 0.134****
**Cu intake (mg/24 h)**				
**Control**	**0.814 ± 0.011**	**0.499 ± 0.015****	**0.559 ± 0.010****	**0.699 ± 0.012****
**AE**	**0.811 ± 0.013**	**0.513 ± 0.012****	**0.564 ± 0.010****	**0.676 ± 0.012****
**Cd_1**	**0.802 ± 0.016**	**0.513 ± 0.008****	**0.564 ± 0.008****	**0.713 ± 0.014****
**Cd_1 + AE**	**0.786 ± 0.009**	**0.522 ± 0.012****	**0.549 ± 0.010****	**0.709 ± 0.021****
**Cd_5**	**0.801 ± 0.010**	**0.520 ± 0.006****	**0.561 ± 0.008****	**0.726 ± 0.025****
**Cd_5 + AE**	**0.815 ± 0.007**	**0.502 ± 0.004****	**0.557 ± 0.004****	**0.698 ± 0.022****

[1] The rats received 0.1% aqueous extract from the berries of *Aronia melanocarpa* (AE) or not and Cd in diet at the concentration of 0, 1, and 5 mg/kg. [2] The study was performed in the last week of the 3rd, 10th, 17th, and 24th month of the experiment. [3] The intake of Zn and Cu was calculated based on these bioelements concentration in the Labofeed diets declared by the manufacturer. The Labofeed H diet (administered throughout the first 3 months of the study) contained 210 mg Zn/kg and 33 mg Cu/kg, whereas the Labofeed B diet (used thereafter), contained 150 mg Zn/kg and 25 mg Cu/kg. Data represent mean ± SE for eight rats (except for seven animals in the AE, Cd_1, and Cd_5 groups after 24 months). $^{**}p < 0.01$ (Anova, Duncan's multiple range test) compared to the intake in the last week of the 3rd month.

Table S4. Main and interactive effects of cadmium (Cd) and the extract from the berries of *Aronia melanocarpa* (AE) on the apparent absorption (Abs_{Zn}), retention in the body (Ret_{Zn}), and faecal (FE_{Zn}) and urinary (UE_{Zn}) excretion of zinc (Zn). [1, 2].

	Main Effect of Cd	Main Effect of AE	Interaction Cd + AE	Main Effect of Cd	Main Effect of AE	Interaction Cd + AE
			**1 mg Cd/kg diet + AE**			
		**3 months**			**10 months**	
Abs_{Zn}	7.91**	27.0***	48.7***	NS	NS	NS
Ret_{Zn}	7.88**	27.0***	48.7***	NS	NS	NS
FE_{Zn}	16.7***	17.9***	34.3***	5.42*	11.1**	NS
UE_{Zn}	-	-	-	-	-	-
		**17 months**			**24 months**	
Abs_{Zn}	-	-	-	-	-	-
Ret_{Zn}	-	-	-	-	-	-
FE_{Zn}	-	-	-	-	-	-
UE_{Zn}	-	-	-	5.06*	4.70*	NS
			**5 mg Cd/kg diet + AE**			
		**3 months**			**10 months**	
Abs_{Zn}	49.6***	NS	NS	-	-	-
Ret_{Zn}	6.36*	NS	NS	-	-	-
FE_{Zn}	50.0***	NS	NS	-	-	-
UE_{Zn}	-	-	-	19.4***	NS	NS
		**17 months**			**24 months**	
Abs_{Zn}	-	-	-	7.81**	5.88*	10.1**
Ret_{Zn}	-	-	-	NS	NS	NS
FE_{Zn}	-	-	-	5.16*	NS	10.9**
UE_{Zn}	-	-	-	NS	NS	7.45*

[1] The rats received 0.1% aqueous AE and Cd in diet at the concentration of 1 or 5 mg/kg. [2] In the case when a one way-analysis of variance (Anova, Duncan's multiple range test) revealed any influence of the co-administration of Cd and AE on the investigated parameter, a two-way analysis of variance (Anova/Manova, test F) was conducted in aim to discern possible interactive and independent impact of Cd and the AE on this parameter. The results of the Anova/Manova analysis are presented as F values and the level of statistical significance (p). F values having $p < 0.05$ were considered statistically significant (* $p < 0.05$, ** $p < 0.01$, *** $p < 0.001$). NS – not statistically significant ($p > 0.05$).

Table S5. Main and interactive effects of cadmium (Cd) and the extract from the berries of *Aronia melanocarpa* (AE) on zinc (Zn) concentration in the serum and tissues of rats exposed to the 1 mg Cd/kg diet. [1, 2].

Tissue Zn concentration	Main Effect of Cd	Main Effect of AE	Interaction Cd + AE	Main Effect of Cd	Main Effect of AE	Interaction Cd + AE
		**3 months**			**10 months**	
Serum	-	-	-	-	-	-
Liver	-	-	-	-	-	-
Kidney	NS	NS	NS	-	-	-
Spleen	-	-	-	-	-	-
Brain	10.8**	11.3**	22.7***	-	-	-
Heart	-	-	-	9.71**	NS	NS
Stomach	35.4***	NS	5.39*	12.2**	9.20**	NS
Duodenum	-	-	-	-	-	-
Bone tissue – femoral diaphysis	-	-	-	NS	NS	NS
Bone tissue – femoral distal epiphysis	-	-	-	-	-	-
Femoral muscle	-	-	-	-	-	-
		**17 months**			**24 months**	
Serum	-	-	-	-	-	-
Liver	7.05*	15.2***	NS	NS	NS	NS
Kidney	-	-	-	NS	13.4**	4.55*
Spleen	9.71**	NS	NS	4.80*	NS	14.1***
Brain	-	-	-	-	-	-

**Heart**	**NS**	**4.59***	**NS**	**-**	**-**	**-**
**Stomach**	**NS**	**NS**	**5.13***	**4.55***	**NS**	**NS**
**Duodenum**	**6.29***	**NS**	**8.37****	**NS**	**5.87***	**NS**
**Bone tissue – femoral diaphysis**	**-**	**-**	**-**	**-**	**-**	**-**
**Bone tissue – femoral distal epiphysis**	**-**	**-**	**-**	**NS**	**NS**	**NS**
**Femoral muscle**	**-**	**-**	**-**	**12.0****	**6.13***	**10.1****

[1] The rats received 0.1% aqueous AE and Cd in diet at the concentration of 1 mg/kg. [2] In the case when a one way-analysis of variance (Anova, Duncan's multiple range test) revealed any influence of the co-administration of Cd and AE on the investigated parameter, a two-way analysis of variance (Anova/Manova, test F) was conducted in aim to discern possible interactive and independent impact of Cd and AE on this parameter. The results of the Anova/Manova analysis are presented as F values and the level of statistical significance (*p*). F values having $p < 0.05$ were considered statistically significant (* $p < 0.05$, ** $p < 0.01$, *** $p < 0.001$). NS – not statistically significant ($p > 0.05$).

Table S6. Main and interactive effects of cadmium (Cd) and the extract from the berries of *Aronia melanocarpa* (AE) on zinc (Zn) concentration in the serum and tissues of rats exposed to the 5 mg Cd/kg diet. [1,2].

**Tissue Zn concentration**	**Main effect of Cd**	**Main effect of AE**	**Interaction Cd + AE**	**Main effect of Cd**	**Main effect of AE**	**Interaction Cd + AE**
		**3 months**			**10 months**	
**Serum**	**NS**	**NS**	**NS**	**-**	**-**	**-**
**Liver**	**-**	**-**	**-**	**-**	**-**	**-**
**Kidney**	**5.06***	**NS**	**NS**	**5.47***	**NS**	**5.68***
**Spleen**	**NS**	**NS**	**7.97****	**4.28***	**NS**	**NS**
**Brain**	**-**	**-**	**-**	**NS**	**NS**	**NS**
**Heart**	**NS**	**12.7****	**NS**	**17.6*****	**NS**	**NS**
**Stomach**	**32.7*****	**-**	**11.6****	**-**	**-**	**-**
**Duodenum**	**-**	**-**	**-**	**-**	**-**	**-**
**Bone tissue – femoral diaphysis**	**NS**	**NS**	**NS**	**NS**	**NS**	**NS**
**Bone tissue – femoral distal epiphysis**	**-**	**-**	**-**	**39.1*****	**9.71****	**19.0*****
**Femoral muscle**	**NS**	**NS**	**9.38****	**-**	**-**	**-**
		**17 months**			**24 months**	
**Serum**	**NS**	**NS**	**NS**	**8.58****	**NS**	**NS**
**Liver**	**-**	**-**	**-**	**NS**	**5.01***	**7.64***
**Kidney**	**27.1*****	**NS**	**NS**	**147.2*****	**NS**	**NS**
**Spleen**	**42.9*****	**NS**	**NS**	**12.2****	**NS**	**11.8****
**Brain**	**-**	**-**	**-**	**NS**	**NS**	**13.5****
**Heart**	**5.29***	**7.77****	**NS**	**-**	**-**	**-**
**Stomach**	**-**	**-**	**-**	**-**	**-**	**34.7*****
**Duodenum**	**23.5*****	**7.95****	**6.52***	**-**	**-**	**-**
**Bone tissue – femoral diaphysis**	**-**	**-**	**-**	**-**	**-**	**-**
**Bone tissue – femoral distal epiphysis**	**-**	**-**	**-**	**-**	**-**	**-**
**Femoral muscle**	**8.28****	**NS**	**NS**	**-**	**-**	**-**

[1] The rats received 0.1% aqueous AE and Cd in diet at the concentration of 5 mg/kg. [2] In the case when a one way-analysis of variance (Anova, Duncan's multiple range test) revealed any influence of the co-administration of Cd and AE on the investigated parameter, a two-way analysis of variance (Anova/Manova, test F) was conducted in aim to discern possible interactive and independent impact of Cd and AE on this parameter. The results of the Anova/Manova analysis are presented as F values and the level of statistical significance (*p*). F values having $p < 0.05$ were considered statistically significant (* $p < 0.05$, ** $p < 0.01$, *** $p < 0.001$). NS – not statistically significant ($p > 0.05$).

Table S7. Main and interactive effects of cadmium (Cd) and the extract from the berries of *Aronia melanocarpa* (AE) on zinc (Zn) content in internal organs. [1,2].

	Main Effect of Cd	Main Effect of AE	Interaction Cd + AE	Main Effect of Cd	Main Effect of AE	Interaction Cd + AE
**1 mg Cd/kg diet + AE**						
	**3 months**			**10 months**		
**Total pool**	NS	NS	NS	-	-	-
**Liver + kidneys**	NS	NS	NS	-	-	-
**Kidney**	-	-	-	-	-	-
**Liver**	4.92*	NS	5.31*	-	-	-
**Spleen**	-	-	-	-	-	-
**Heart**	-	-	-	-	-	-
**Brain**	9.51**	7.22*	NS	-	-	-
	**17 months**			**24 months**		
**Total pool**	-	-	-	NS	NS	NS
**Liver + kidneys**	-	-	-	10.5**	NS	NS
**Kidney**	-	-	-	NS	NS	NS
**Liver**	-	-	-	-	-	-
**Spleen**	NS	9.11**	NS	-	-	-
**Heart**	-	-	-	NS	5.00*	NS
**Brain**	-	-	-	NS	NS	5.42*
**5 mg Cd/kg diet + AE**						
	**3 months**			**10 months**		
**Total pool**	-	-	-	7.92**	NS	11.8**
**Liver + kidneys**	-	-	-	NS	NS	NS
**Kidney**	-	-	-	15.6***	NS	NS
**Liver**	-	-	-	5.97*	NS	12.6**
**Spleen**	NS	NS	NS	-	-	-
**Heart**	NS	6.65*	NS	-	-	-
**Brain**	NS	NS	NS	NS	NS	NS
	**17 months**			**24 months**		
**Total pool**	-	-	-	NS	6.30*	NS
**Liver + kidneys**	-	-	-	NS	6.59*	NS
**Kidney**	5.13*	NS	NS	NS	NS	NS
**Liver**	-	-	-	NS	6.02*	NS
**Spleen**	-	-	-	-	-	-
**Heart**	-	-	-	-	-	-
**Brain**	-	-	-	4.59*	NS	NS

[1] The rats received 0.1% aqueous AE and Cd in diet at the concentration of 1 or 5 mg/kg. [2] In the case when a one way-analysis of variance (Anova, Duncan's multiple range test) revealed any influence of the co-administration of Cd and AE on the investigated parameter, a two-way analysis of variance (Anova/Manova, test F) was conducted in aim to discern possible interactive and independent impact of Cd and AE on this parameter. The results of the Anova/Manova analysis are presented as F values and the level of statistical significance (p). F values having $p < 0.05$ were considered statistically significant (* $p < 0.05$, ** $p < 0.01$, *** $p < 0.001$). NS—not statistically significant ($p > 0.05$).

Table S8. Main and interactive effects of cadmium (Cd) and the extract from the berries of *Aronia melanocarpa* (AE) on the apparent absorption (Abs_{Cu}), retention in the body (Ret_{Cu}), and faecal (FE_{Cu}) and urinary (UE_{Cu}) excretion of copper (Cu). [1,2].

	Main effect of Cd	Main effect of AE	Interaction Cd + AE	Main effect of Cd	Main effect of AE	Interaction Cd + AE
			**1 mg Cd/kg diet +AE**			
		**3 months**			**10 months**	
**Abs_{Cu}**	**16.9^{***}**	**6.44^{*}**	**14.8^{***}**	**NS**	**11.0^{**}**	**NS**
**Ret_{Cu}**	**16.9^{***}**	**6.36^{*}**	**14.7^{***}**	**NS**	**14.7^{***}**	**NS**
**FE_{Cu}**	**9.59^{**}**	**4.72^{*}**	**11.6^{**}**	**NS**	**29.0^{***}**	**NS**
**UE_{Cu}**	**-**	**-**	**-**	**-**	**-**	**-**
		**17 months**			**24 months**	
**Abs_{Cu}**	**-**	**-**	**-**	**5.20^{*}**	**NS**	**NS**
**Ret_{Cu}**	**-**	**-**	**-**	**5.40^{*}**	**NS**	**NS**
**FE_{Cu}**	**-**	**-**	**-**	**6.24^{*}**	**NS**	**NS**
**UE_{Cu}**	**NS**	**NS**	**NS**	**6.73^{*}**	**NS**	**4.63^{*}**
			**5 mg Cd/kg diet + AE**			
		**3 months**			**10 months**	
**Abs_{Cu}**	**6.40^{*}**	**NS**	**NS**	**-**	**-**	**-**
**Ret_{Cu}**	**6.36^{*}**	**NS**	**NS**	**-**	**-**	**-**
**FE_{Cu}**	**5.83^{*}**	**NS**	**NS**	**-**	**-**	**-**
**UE_{Cu}**	**-**	**-**	**-**	**-**	**-**	**-**
		**17 months**			**24 months**	
**Abs_{Cu}**	**-**	**-**	**-**	**-**	**-**	**-**
**Ret_{Cu}**	**-**	**-**	**-**	**-**	**-**	**-**
**FE_{Cu}**	**-**	**-**	**-**	**-**	**-**	**-**
**UE_{Cu}**	**-**	**-**	**-**	**6.60^{*}**	**8.61^{**}**	**4.57^{*}**

[1] The rats received 0.1% aqueous AE and Cd in diet at the concentration of 1 or 5 mg/kg. [2] In the case when a one way-analysis of variance (Anova, Duncan's multiple range test) revealed any influence of the co-administration of Cd and AE on the investigated parameter, a two-way analysis of variance (Anova/Manova, test F) was conducted in aim to discern possible interactive and independent impact of Cd and AE on this parameter. The results of the Anova/Manova analysis are presented as F values and the level of statistical significance (p). F values having $p < 0.05$ were considered statistically significant (* $p < 0.05$, ** $p < 0.01$, *** $p < 0.001$). NS – not statistically significant ($p > 0.05$).

Table S9. Main and interactive effects of cadmium (Cd) and the extract from the berries of *Aronia melanocarpa* (AE) on copper (Cu) concentration in the serum and tissues of rats exposed to the 1 mg Cd/kg diet. [1,2].

Tissue Cu concentration	Main effect of Cd	Main effect of AE	Interaction Cd + AE	Main effect of Cd	Main effect of AE	Interaction Cd + AE
		**3 months**			**10 months**	
**Serum**	**NS**	**18.8^{***}**	**9.44^{**}**	**-**	**-**	**-**
**Liver**	**-**	**-**	**-**	**-**	**-**	**-**
**Kidney**	**7.18^{*}**	**NS**	**11.5^{**}**	**32.5^{***}**	**NS**	**NS**
**Spleen**	**-**	**-**	**-**	**-**	**-**	**-**
**Brain**	**NS**	**4.62^{*}**	**14.3^{***}**	**NS**	**4.69^{*}**	**7.96^{**}**
**Heart**	**9.35^{**}**	**NS**	**46.0^{***}**	**-**	**-**	**-**
**Stomach**	**NS**	**7.05^{*}**	**9.41^{**}**	**NS**	**13.6^{***}**	**NS**
**Duodenum**	**-**	**-**	**-**	**-**	**-**	**-**

**Bone tissue – femoral diaphysis**	-	-	-	-	-	-
**Bone tissue – femoral distal epiphysis**	NS	NS	NS	-	-	-
**Femoral muscle**	NS	NS	5.32*	-	-	-
		**17 months**			**24 months**	
**Serum**	15.4***	NS	NS	-	-	-
**Liver**	NS	5.87*	10.3**	-	-	-
**Kidney**	56.8***	5.87*	37.9***	8.42**	NS	NS
**Spleen**	NS	NS	NS	NS	NS	10.2**
**Brain**	-	-	-	NS	5.55*	NS
**Heart**	-	-	-	-	-	-
**Stomach**	17.0***	NS	NS	-	-	-
**Duodenum**	NS	11.4**	NS	NS	11.6**	6.95*
**Bone tissue – femoral diaphysis**	-	-	-	-	-	-
**Bone tissue – femoral distal epiphysis**	NS	NS	NS	-	-	-
**Femoral muscle**	-	-	-	-	-	-

[1] The rats received 0.1% aqueous AE and Cd in diet at the concentration of 1 mg/kg. [2] In the case when a one way-analysis of variance (Anova, Duncan's multiple range test) revealed any influence of the co-administration of Cd and AE on the investigated parameter, a two-way analysis of variance (Anova/Manova, test F) was conducted in aim to discern possible interactive and independent impact of Cd and AE on this parameter. The results of the Anova/Manova analysis are presented as F values and the level of statistical significance (p). F values having $p < 0.05$ were considered statistically significant (* $p < 0.05$, ** $p < 0.01$, *** $p < 0.001$). NS – not statistically significant ($p > 0.05$).

Table S10. Main and interactive effects of cadmium (Cd) and the extract from the berries of *Aronia melanocarpa* (AE) on copper (Cu) concentration in the serum and tissues of rats exposed to the 5 mg Cd/kg diet. [1, 2].

Tissue Cu concentration	Main effect of Cd	Main effect of AE	Interaction Cd + AE	Main effect of Cd	Main effect of AE	Interaction Cd + AE
		**3 months**			**10 months**	
**Serum**	5.32*	30.3***	NS	NS	NS	NS
**Liver**	-	-	-	-	-	-
**Kidney**	NS	28.6***	14.1***	24.7***	48.3***	45.2***
**Spleen**	6.69*	NS	NS	-	-	-
**Brain**	-	-	-	14.6***	NS	NS
**Heart**	NS	23.5***	NS	-	-	-
**Stomach**	NS	5.9*	5.76*	4.55*	NS	NS
**Duodenum**	-	-	-	-	-	-
**Bone tissue – femoral diaphysis**	17.96***	NS	NS	NS	NS	NS
**Bone tissue – femoral distal epiphysis**	13.5**	NS	NS	-	-	-
**Femoral muscle**	-	-	-	-	-	-
		**17 months**			**24 months**	
**Serum**	NS	NS	NS	NS	17.3***	12.4**

Liver	-	-	-	5.47*	NS	NS
Kidney	NS	NS	8.59**	-	-	-
Spleen	-	-	-	NS	14.6***	5.28*
Brain	-	-	-	-	-	-
Heart	-	-	-	NS	NS	NS
Stomach	29.9***	NS	NS	-	-	-
Duodenum	15.6***	10.5**	NS	NS	NS	10.3***
Bone tissue – femoral diaphysis	-	-	-	-	-	-
Bone tissue – femoral distal epiphysis	NS	NS	NS	NS	NS	NS
Femoral muscle	-	-	-	-	-	-

[1] The rats received 0.1% aqueous AE and Cd in diet at the concentration of 5 mg/kg. [2] In the case when a one way-analysis of variance (Anova, Duncan's multiple range test) revealed any influence of the co-administration of Cd and AE on the investigated parameter, a two-way analysis of variance (Anova/Manova, test F) was conducted in aim to discern possible interactive and independent impact of Cd and AE on this parameter. The results of the Anova/Manova analysis are presented as F values and the level of statistical significance (p). F values having $p < 0.05$ were considered statistically significant (* $p < 0.05$, ** $p < 0.01$, *** $p < 0.001$). NS – not statistically significant ($p > 0.05$).

Table S11. Main and interactive effects of cadmium (Cd) and the extract from the berries of *Aronia melanocarpa* (AE) on copper (Cu) content in internal organs. [1, 2].

	Main effect of Cd	Main effect of AE	Interaction Cd + AE	Main effect of Cd	Main effect of AE	Interaction Cd + AE
			1 mg Cd/kg diet +AE			
		3 months			10 months	
Total pool	7.60*	NS	NS	-	-	-
Liver + kidneys	NS	NS	NS	-	-	-
Kidney	14.4***	NS	10.6**	13.0**	NS	NS
Liver	-	-	-	-	-	-
Spleen	-	-	-	-	-	-
Heart	NS	27.1***	NS	-	-	-
Brain	12.8**	NS	28.3***	NS	NS	6.77*
		17 months			24 months	
Total pool	7.50*	NS	NS	6.88*	NS	NS
Liver + kidneys	NS	NS	NS	12.9**	NS	NS
Kidney	21.6***	NS	NS	-	-	-
Liver	NS	NS	NS	-	-	-
Spleen	-	-	-	4.64*	4.71*	NS
Heart	-	-	-	5.05*	4.34*	NS
Brain	-	-	-	NS	10.1**	NS
			5 mg Cd/kg diet +AE			
		3 months			10 months	
Total pool	NS	NS	NS	12.0**	7.45*	16.0***
Liver + kidneys	NS	NS	NS	NS	NS	NS
Kidney	NS	41.2***	26.7***	38.0***	49.1***	54.1***
Liver	-	-	-	NS	NS	8.41**

**Spleen**	**-**	**-**	**-**	**-**	**-**	
**Heart**	**NS**	**27.1*****	**NS**	**-**	**-**	**-**
**Brain**	**-**	**-**	**-**	**14.6*****	**NS**	**NS**
		**17 months**			**24 months**	
**Total pool**	**-**	**-**	**-**	**NS**	**6.39***	**NS**
**Liver + kidneys**	**-**	**-**	**-**	**NS**	**6.98***	**NS**
**Kidney**	**-**	**-**	**-**	**-**	**-**	**-**
**Liver**	**-**	**-**	**-**	**NS**	**7.02***	**NS**
**Spleen**	**-**	**-**	**-**	**-**	**-**	**-**
**Heart**	**-**	**-**	**-**	**-**	**-**	**-**
**Brain**	**-**	**-**	**-**	**-**	**-**	**-**

[1] The rats received 0.1% aqueous AE and Cd in diet at the concentration of 1 mg/kg. [2] In the case when a one way-analysis of variance (Anova, Duncan's multiple range test) revealed any influence of the co-administration of Cd and AE on the investigated parameter, a two-way analysis of variance (Anova/Manova, test F) was conducted in aim to discern possible interactive and independent impact of Cd and AE on this parameter. The results of the Anova/Manova analysis are presented as F values and the level of statistical significance (p). F values having $p < 0.05$ were considered statistically significant (* $p < 0.05$, ** $p < 0.01$, *** $p < 0.001$). NS – not statistically significant ($p > 0.05$).

Table S12. Main and interactive effects of cadmium (Cd) and the extract from the berries of *Aronia melanocarpa* (AE) on metallothionein (MT) concentration in the liver and the degree of zinc (Zn), copper (Cu), and Cd binding to this protein. [1,2].

	**Main effect of Cd**	**Main effect of AE**	**Interaction Cd + AE**	**Main effect of Cd**	**Main effect of AE**	**Interaction Cd + AE**
			**1 mg Cd/kg diet + AE**			
		**3 months**			**10 months**	
**MT**	**145.5*****	**7.20***	**4.86***	**20.8*****	**24.5*****	**19.0*****
**Zn/(MT x 7)**	**11.3****	**17.6*****	**NS**	**4.74***	**22.4*****	**12.4****
**Cu/(MT x 12)**	**5.96***	**NS**	**NS**	**NS**	**16.7*****	**13.1****
**Cd/(MT x 7)**	**-**	**-**	**-**	**94.5*****	**22.9*****	**23.4*****
**Me/(Me-MT)**	**NS**	**NS**	**NS**	**4.54***	**22.1*****	**13.1***
		**17 months**			**24 months**	
**MT**	**35.3*****	**9.63****	**35.3*****	**10.7****	**NS**	**16.4*****
**Zn/(MT x 7)**	**34.0*****	**NS**	**22.2*****	**5.95***	**NS**	**7.86****
**Cu/(MT x 12)**	**15.9*****	**NS**	**28.2*****	**9.48****	**NS**	**14.6*****
**Cd/(MT x 7)**	**-**	**-**	**-**	**-**	**-**	**-**
**Me/(Me-MT)**	**32.4*****	**NS**	**23.2*****	**6.10***	**NS**	**13.1****
			**5 mg Cd/kg diet + AE**			
		**3 months**			**10 months**	
**MT**	**15.6*****	**21.2*****	**6.88***	**36.7*****	**13.4****	**6.97***
**Zn/(MT x 7)**	**11.2****	**13.9*****	**NS**	**53.0*****	**12.4****	**NS**
**Cu/(MT x 12)**	**10.6****	**8.33****	**NS**	**51.6*****	**8.04****	**4.49***
**Cd/(MT x 7)**	**74.1*****	**7.67****	**7.22***	**211.3*****	**13.3****	**13.4****
**Me/(Me-MT)**	**51.7*****	**15.9*****	**11.4****	**23.9*****	**NS**	**5.24***
		**17 months**			**24 months**	
**MT**	**44.6*****	**54.8*****	**115.2*****	**27.4*****	**26.8*****	**68.3*****
**Zn/(MT x 7)**	**9.06****	**6.69***	**61.1*****	**4.95***	**NS**	**37.7*****
**Cu/(MT x 12)**	**4.94***	**6.94***	**44.65*****	**10.11****	**NS**	**33.3*****
**Cd/(MT x 7)**	**724.3*****	**79.3*****	**80.1*****	**209.3*****	**38.6*****	**39.1*****
**Me/(Me-MT)**	**13.1****	**NS**	**13.4****	**12.5****	**NS**	**15.5*****

[1] The rats received 0.1% aqueous AE and Cd in diet at the concentration of 1 or 5 mg/kg. [2] In the case when a one way-analysis of variance (Anova, Duncan's multiple range test) revealed any influence of the co-administration of Cd and AE on the investigated parameter, a two-way analysis of variance (Anova/Manova, test F) was conducted in aim to discern possible interactive and independent impact of Cd and the AE on this parameter. The results of the Anova/Manova analysis are presented as F values and the level of statistical significance (p). F values having $p < 0.05$ were considered statistically significant (* $p < 0.05$, ** $p < 0.01$, *** $p < 0.001$). NS – not statistically significant ($p > 0.05$). Zn/(MT x 7), the pool of MT-unbound Zn; Cu/(MT x 12), the pool of MT-unbound Cu; Cd/(MT x 7), the pool of MT-unbound Cd; Me/(Me-MT), the pool of MT-unbound metals (Zn, Cu and Cd).

Table S13. Main and interactive effects of cadmium (Cd) and the extract from the berries of *Aronia melanocarpa* (AE) on metallothionein (MT) concentration in the kidney and the degree of zinc (Zn), copper (Cu), and Cd binding to this protein. [1, 2].

	Main effect of Cd	Main effect of AE	Interaction Cd + AE	Main effect of Cd	Main effect of AE	Interaction Cd + AE
		1 mg Cd/kg diet + AE				
		3 months			10 months	
MT	NS	8.91**	8.22**	7.54*	NS	7.41*
Zn/(MT x 7)	NS	7.78**	NS	12.8**	NS	10.7**
Cu/(MT x 12)	NS	9.54**	-	18.4***	NS	8.95**
Cd/(MT x 7)	186.5***	NS	NS	313.8***	NS	NS
Me/(Me-MT)	NS	8.46**	4.66*	12.1**	NS	10.6**
		17 months			24 months	
MT	4.24*	18.2***	11.6**	40.9***	7.45*	NS
Zn/(MT x 7)	NS	15.9***	7.65**	29.5***	NS	NS
Cu/(MT x 12)	12.7**	19.6***	26.3***	6.81*	NS	NS
Cd/(MT x 7)	228***	20.4***	19.0***	202.3***	7.73**	6.05*
Me/(Me-MT)	NS	17.2***	10.3**	25.0***	NS	NS
		5 mg Cd/kg diet + AE				
		3 months			10 months	
MT	NS	13.3**	12.5**	33.5***	NS	5.22*
Zn/(MT x 7)	NS	10.4**	5.14*	36.7***	NS	12.3**
Cu/(MT x 12)	-	-	-	19.6***	8.90**	NS
Cd/(MT x 7)	284.8***	14.1***	14.1***	549.7***	NS	NS
Me/(Me-MT)	10.6**	13.5**	NS	49.8***	12.3**	NS
		17 months			24 months	
MT	22.1***	32.0***	23.3***	92.0***	12.2**	5.93*
Zn/(MT x 7)	NS	7.42*	NS	59.5***	8.62**	NS
Cu/(MT x 12)	16.6***	20.7***	29.4***	1411.9***	10.2**	12.6**
Cd/(MT x 7)	395.3***	21.8***	21.5***	380.1***	5.12*	4.54*
Me/(Me-MT)	6.50*	7.51*	62.4***	41.1***	NS	11.7**

[1] The rats received 0.1% aqueous AE and Cd in diet at the concentration of 1 or 5 mg/kg. [2] In the case when a one way-analysis of variance (Anova, Duncan's multiple range test) revealed any influence of the co-administration of Cd and AE on the investigated parameter, a two-way analysis of variance (Anova/Manova, test F) was conducted in aim to discern possible interactive and independent impact of Cd and AE on this parameter. The results of the Anova/Manova analysis are presented as F values and the level of statistical significance (p). F values having $p < 0.05$ were considered statistically significant (* $p < 0.05$, ** $p < 0.01$, *** $p < 0.001$). NS – not statistically significant ($p > 0.05$). Zn/(MT x 7), the pool of MT-unbound Zn; Cu/(MT x 12), the pool of MT-unbound Cu; Cd/(MT x 7), the pool of MT-unbound Cd; Me/(Me-MT), the pool of MT-unbound metals (Zn, Cu and Cd).

Table S14. Main and interactive effects of cadmium (Cd) and the extract from the berries of *Aronia melanocarpa* (AE) on metallothionein (MT) concentration in the duodenum and the degree of zinc (Zn), copper (Cu), and Cd binding to this protein. [1, 2].

	Main effect of Cd	Main effect of AE	Interaction Cd + AE	Main effect of Cd	Main effect of AE	Interaction Cd + AE
			**1 mg Cd/kg diet + AE**			
		**3 months**			**10 months**	
**MT**	NS	6.51^{*}	27.0^{***}	27.9^{***}	NS	NS
**Zn/(MT x 7)**	NS	NS	12.1^{**}	29.3^{***}	NS	NS
**Cu/(MT x 12)**	NS	NS	11.1^{**}	26.3^{***}	NS	NS
**Cd/(MT x 7)**	272.7^{***}	28.5^{***}	59.3^{***}	170.4^{***}	10.0^{**}	11.1^{**}
**Me/(Me-MT)**	NS	NS	12.1^{*}	28.7^{***}	NS	NS
		**17 months**			**24 months**	
**MT**	NS	13.9^{**}	14.0^{***}	63.9^{***}	NS	8.18^{**}
**Zn/(MT x 7)**	NS	NS	26.3^{***}	38.2^{***}	NS	NS
**Cu/(MT x 12)**	NS	4.22^{*}	22.3^{***}	35.5^{***}	NS	NS
**Cd/(MT x 7)**	NS	4.22^{*}	22.3^{***}	82.3^{***}	NS	4.95^{*}
**Me/(Me-MT)**	NS	NS	26.4^{***}	37.9^{***}	NS	NS
			**5 mg Cd/kg diet + AE**			
		**3 months**			**10 months**	
**MT**	8.83^{**}	NS	12.6^{**}	59.4^{***}	9.39^{**}	9.72^{**}
**Zn/(MT x 7)**	10.4^{**}	NS	7.16^{*}	24.6^{***}	NS	5.16^{*}
**Cu/(MT x 12)**	8.17^{**}	NS	5.45^{*}	29.8^{***}	NS	5.08^{*}
**Cd/(MT x 7)**	353.0^{***}	14.4^{***}	20.2^{***}	162.9^{***}	12.92^{**}	13.4^{**}
**Me/(Me-MT)**	9.89^{**}	NS	7.21^{*}	23.9^{***}	NS	5.24^{*}
		**17 months**			**24 months**	
**MT**	8.76^{**}	10.3^{**}	10.4^{**}	232.0^{***}	22.01^{***}	18.7^{***}
**Zn/(MT x 7)**	13.7^{***}	NS	13.3^{**}	13.4^{**}	NS	15.3^{***}
**Cu/(MT x 12)**	3860.5^{***}	6.38^{*}	NS	5688.4^{***}	NS	NS
**Cd/(MT x 7)**	368.0^{***}	10.3^{**}	10.0^{**}	148.4^{***}	20.6^{***}	21.5^{***}
**Me/(Me-MT)**	13.1^{**}	NS	13.4^{**}	12.5^{**}	NS	15.5^{***}

[1] The rats received 0.1% aqueous AE and Cd in diet at the concentration of 1 or 5 mg/kg. [2] In the case when a one way-analysis of variance (Anova, Duncan's multiple range test) revealed any influence of the co-administration of Cd and AE on the investigated parameter, a two-way analysis of variance (Anova/Manova, test F) was conducted in aim to discern possible interactive and independent impact of Cd and AE on this parameter. The results of the Anova/Manova analysis are presented as F values and the level of statistical significance (p). F values having $p < 0.05$ were considered statistically significant (* $p < 0.05$, ** $p < 0.01$, *** $p < 0.001$). NS – not statistically significant ($p > 0.05$). Zn/(MT x 7), the pool of MT-unbound Zn; Cu/(MT x 12), the pool of MT-unbound Cu; Cd/(MT x 7), the pool of MT-unbound Cd; Me/(Me-MT), the pool of MT-unbound metals (Zn, Cu and Cd).

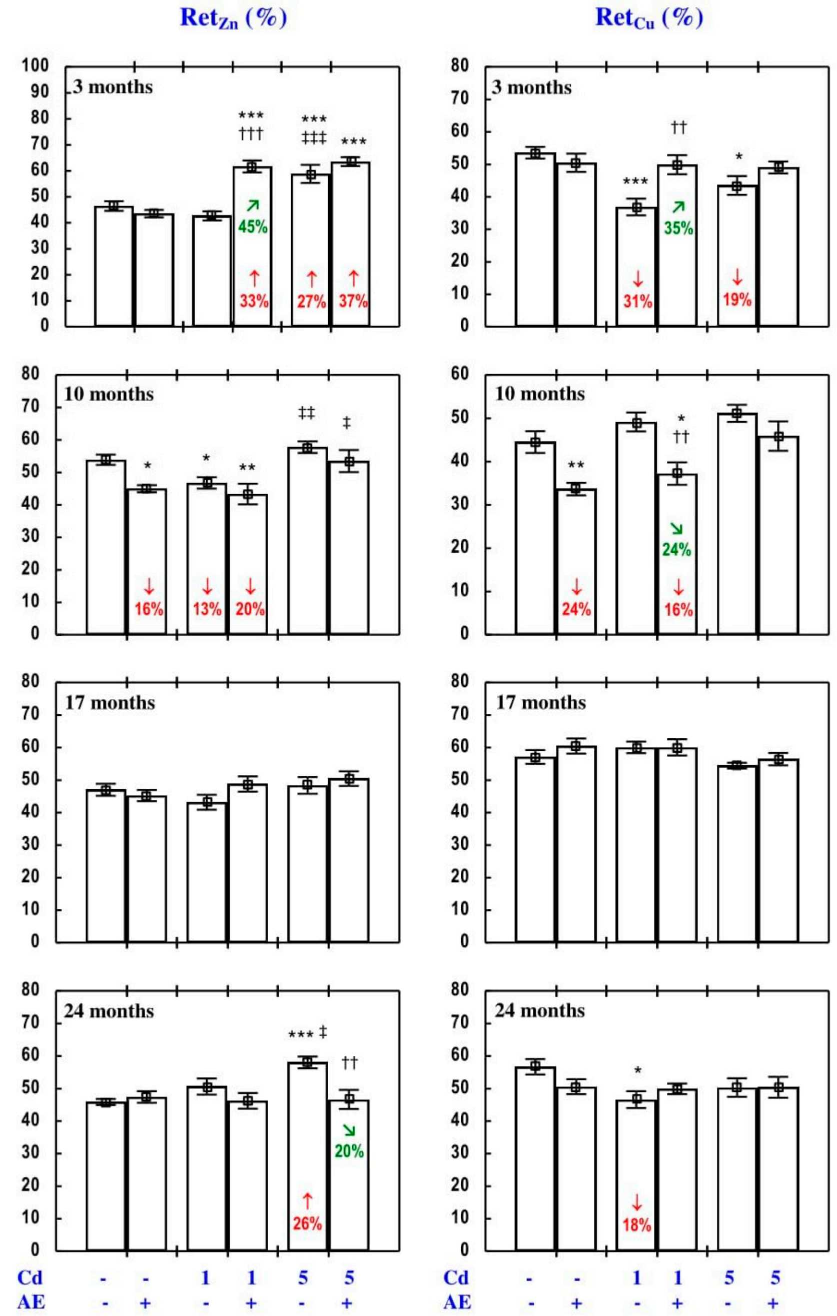

Figure S1. The body retention of zinc (Ret_{Zn}) and copper (Ret_{Cu}) in particular experimental groups. The rats received cadmium (Cd) in diet at the concentration of 0, 1, and 5 mg/kg and/or 0.1% extract from the berries of *Aronia melanocarpa* (AE; "+", received; "-", not received). Data represent mean ± SE for eight rats (except for seven animals in the AE, Cd_1, and Cd_5 groups after 24 months). Statistically significant differences (Anova, Duncan's multiple range test): * $p < 0.05$, ** $p < 0.01$, *** $p < 0.001$ vs. control group; †† $p < 0.01$, ††† $p < 0.001$ vs. respective group receiving Cd alone; ‡ $p < 0.05$, ‡‡ $p < 0.01$, ‡‡‡ $p < 0.001$ vs. respective group receiving the 1 mg Cd/kg diet (alone or with AE) are marked. Numerical values in bars indicate percentage changes compared to the control group (↓, decrease; ↑, increase) or the respective group receiving Cd alone (↘, decrease; ↘, increase).

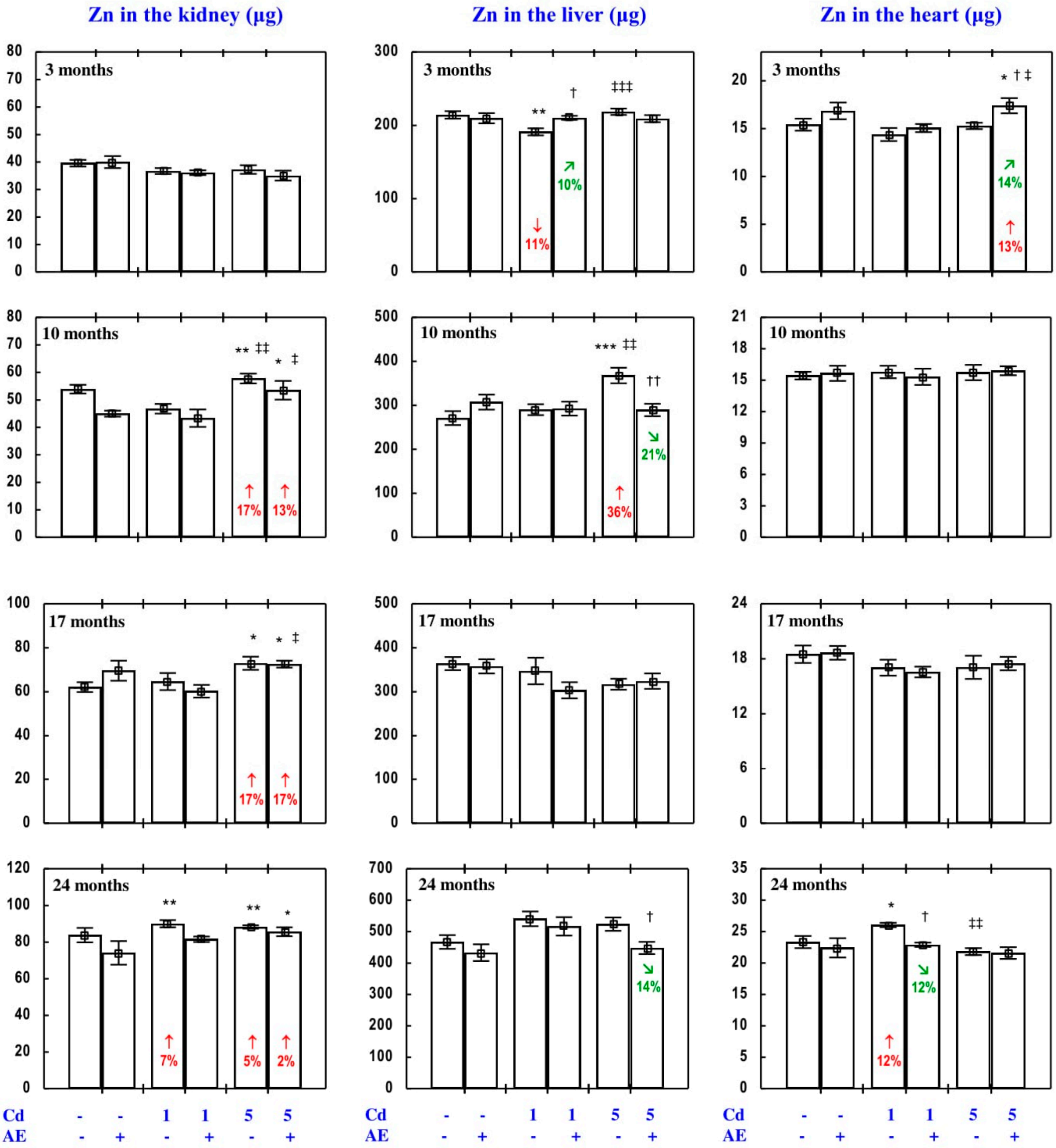

Figure S2. Zinc (Zn) content in the kidney, liver, and heart in particular experimental groups. The rats received cadmium (Cd) in diet at the concentration of 0, 1, and 5 mg/kg and/or 0.1% extract from the berries of *Aronia melanocarpa* (AE; "+", received; "-", not received). Data represent mean ± SE for eight rats (except for seven animals in the AE, Cd_1, and Cd_5 groups after 24 months). Statistically significant differences (Anova, Duncan's multiple range test): * $p < 0.05$, ** $p < 0.01$, *** $p < 0.001$ vs. control group; † $p < 0.05$, †† $p < 0.01$ vs. respective group receiving Cd alone; ‡ $p < 0.05$, ‡‡ $p < 0.01$, ‡‡‡ $p < 0.001$ vs. respective group receiving the 1 mg Cd/kg diet (alone or with AE) are marked. Numerical values in bars indicate percentage changes compared to the control group (↓, decrease; ↑, increase) or the respective group receiving Cd alone (↘, decrease; ↗, increase).

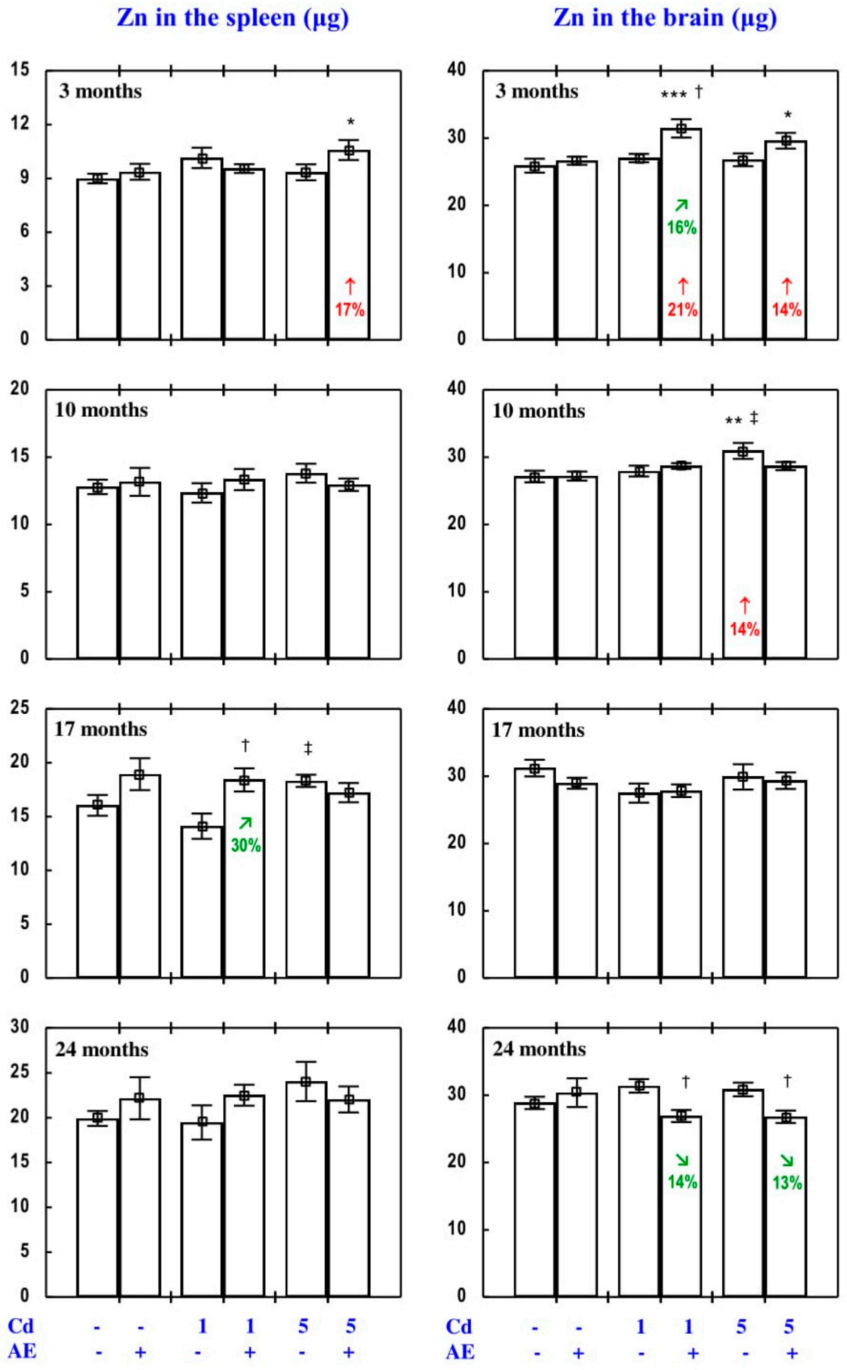

Figure S3. Zinc (Zn) content in the spleen and brain in particular experimental groups. The rats received cadmium (Cd) in diet at the concentration of 0, 1, and 5 mg/kg and/or 0.1% extract from the berries of *Aronia melanocarpa* (AE; "+", received; "-", not received). Data represent mean ± SE for eight rats (except for seven animals in the AE, Cd_1, and Cd_5 groups after 24 months). Statistically significant differences (Anova, Duncan's multiple range test): * $p < 0.05$, ** $p < 0.01$, *** $p < 0.001$ vs. control group; † $p < 0.05$ vs. respective group receiving Cd alone; ‡ $p < 0.05$ vs. respective group receiving the 1 mg Cd/kg diet (alone or with AE) are marked. Numerical values in bars indicate percentage changes compared to the control group (↑, increase) or the respective group receiving Cd alone (↘, decrease; ↗, increase).

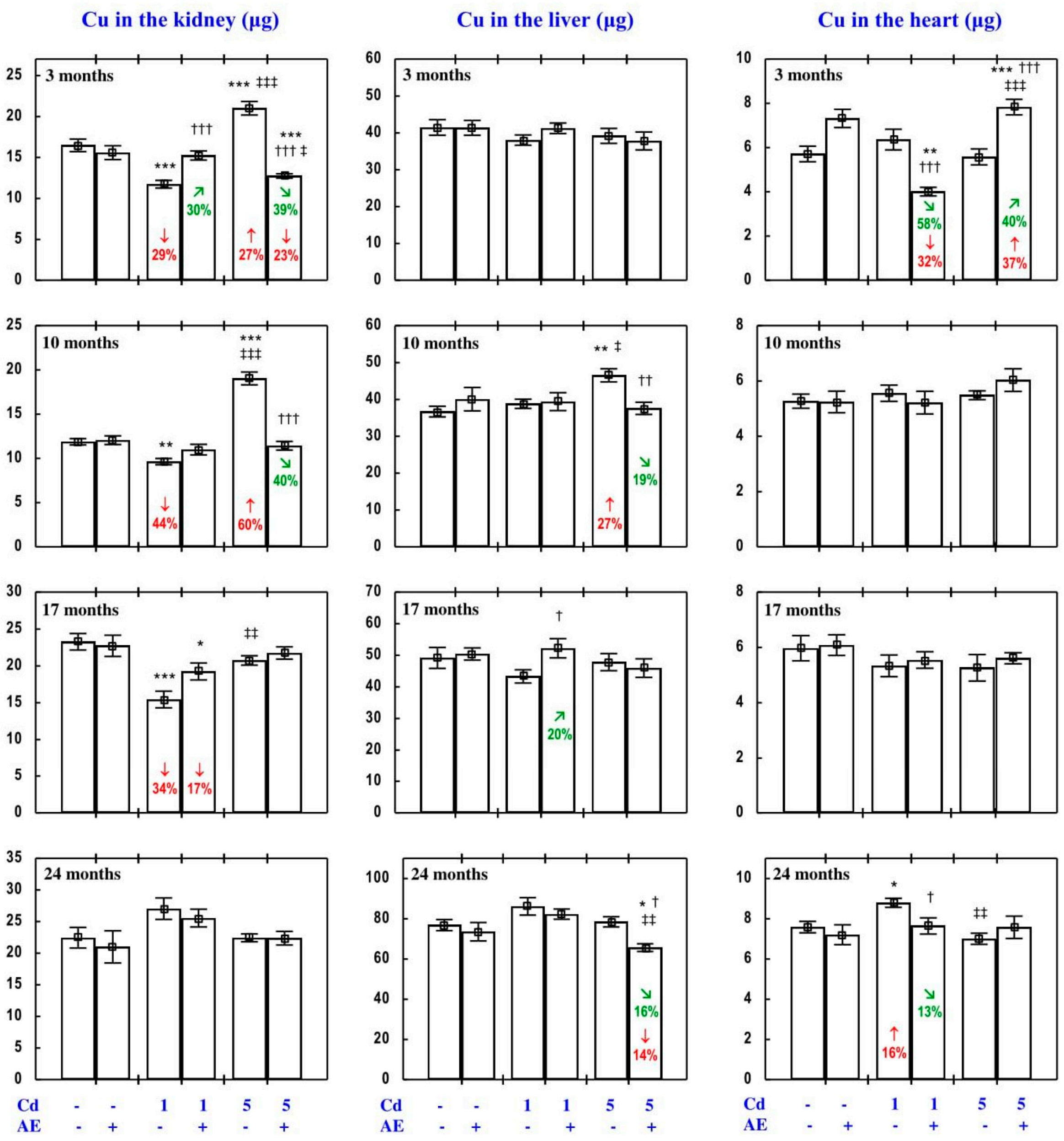

Figure S4. Copper (Cu) content in the kidney, liver, and heart in particular experimental groups. The rats received cadmium (Cd) in diet at the concentration of 0, 1, and 5 mg/kg and/or 0.1% extract from the berries of *Aronia melanocarpa* (AE; "+", received; "-", not received). Data represent mean ± SE for eight rats (except for seven animals in the AE, Cd_1, and Cd_5 groups after 24 months). Statistically significant differences (Anova, Duncan's multiple range test): * $p < 0.05$, ** $p < 0.01$, *** $p < 0.001$ vs. control group; † $p < 0.05$, †† $p < 0.01$, ††† $p < 0.001$ vs. respective group receiving Cd alone; ‡ $p < 0.05$, ‡‡ $p < 0.01$, ‡‡‡ $p < 0.001$ vs. respective group receiving 1 mg Cd/kg diet (alone or with AE) are marked. Numerical values in bars indicate percentage changes compared to the control group (↓, decrease; ↑, increase) or the respective group receiving Cd alone (↘, decrease; ↗, increase).

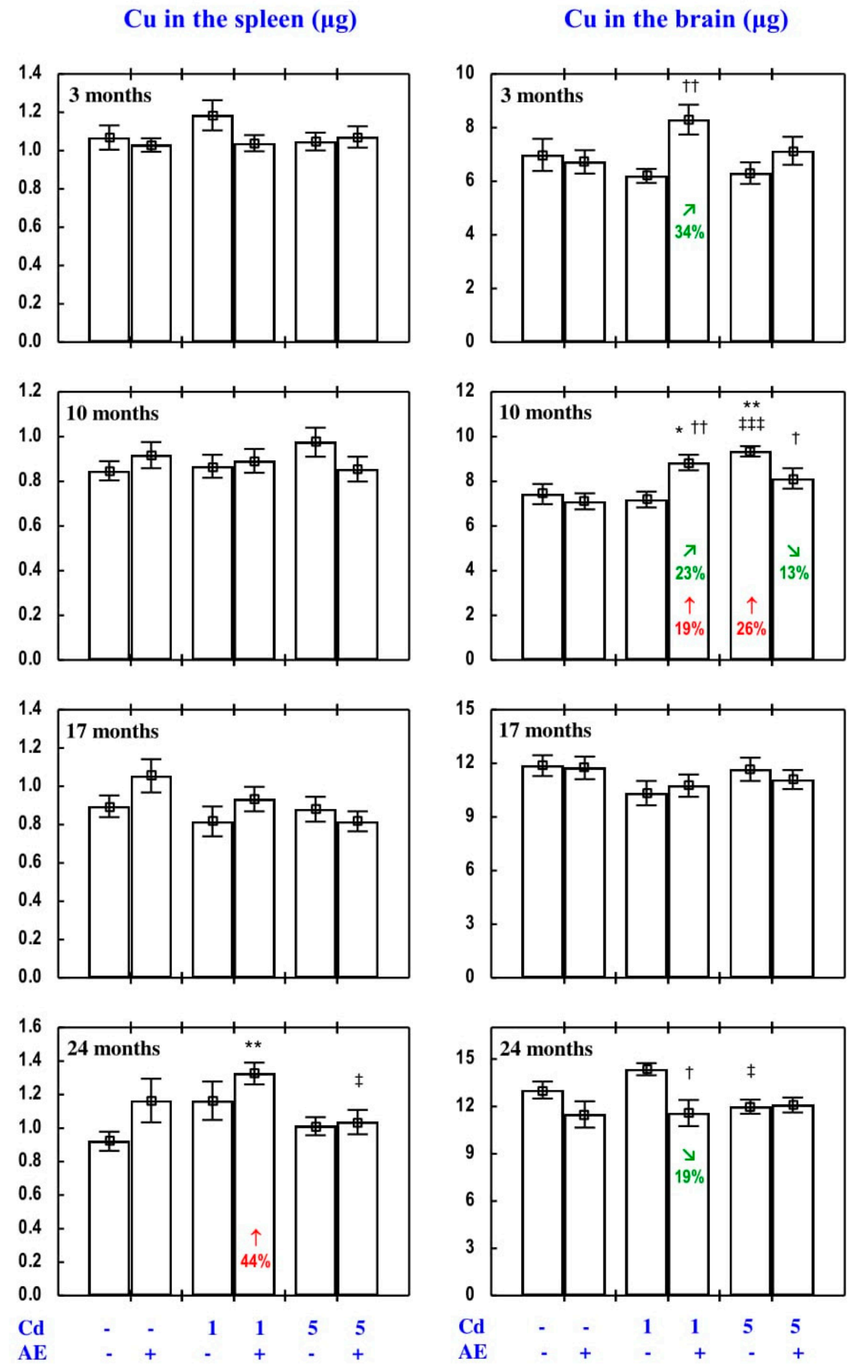

Figure S5. Copper (Cu) content in the spleen and brain in particular experimental groups. The rats received cadmium (Cd) in diet at the concentration of 0, 1, and 5 mg/kg and/or 0.1% extract from the berries of *Aronia melanocarpa* (AE; "+", received; "-", not received). Data represent mean ± SE for eight rats (except for seven animals in the AE, Cd_1, and Cd_5 groups after 24 months). Statistically significant differences (Anova, Duncan's multiple range test): * $p < 0.05$, ** $p < 0.01$ vs. control group; † $p < 0.05$, †† $p < 0.01$ vs. respective group receiving Cd alone; ‡ $p < 0.05$, ‡‡‡ $p < 0.001$ vs. respective group receiving 1 mg Cd/kg diet (alone or with AE) are marked. Numerical values in bars indicate percentage changes compared to the control group (↓, decrease; ↑, increase) or the respective group receiving Cd alone (↘, decrease; ↗, increase).
